# Liverworts show a globally consistent mid‐elevation richness peak

**DOI:** 10.1002/ece3.9862

**Published:** 2023-03-23

**Authors:** Karola Maul, Yu‐Mei Wei, Eka Aditya Putri Iskandar, Sahut Chantanaorrapint, Boon‐Chuan Ho, Dietmar Quandt, Michael Kessler

**Affiliations:** ^1^ Nees Institute for Biodiversity of Plants University of Bonn Bonn Germany; ^2^ Guangxi Key Laboratory of Plant Conservation and Restoration Ecology in Karst Terrain, Guangxi Institute of Botany Guangxi Zhuang Autonomous Region and Chinese Academy of Sciences Guilin China; ^3^ Understanding Evolution Research Group Naturalis Biodiversity Centre Leiden The Netherlands; ^4^ Institute of Biology Leiden, Faculty of Science Leiden University Leiden The Netherlands; ^5^ Cibodas Botanic Garden National Research and Innovation Agency (BRIN) Bandung Indonesia; ^6^ Division of Biological Sciences, Faculty of Science Prince of Songkla University Hat Yai Thailand; ^7^ Singapore Botanic Gardens National Parks Board Singapore Republic of Singapore; ^8^ Systematic and Evolutionary Botany University of Zurich Zurich Switzerland

**Keywords:** climatic predictors, diversity gradients, elevation, liverworts

## Abstract

The study of elevational gradients allows to draw conclusions on the factors and mechanisms determining patterns in species richness distribution. Several earlier studies investigated liverwort diversity on single or few elevational transects. However, a comprehensive survey of the elevational distribution patterns of liverwort richness and their underlying factors is lacking so far. This study's purpose was to fill this gap by compiling an extensive data set of liverwort elevational patterns encompassing a broad diversity of mountains and mountain ranges around the world. Using polynomial regression analyses, we found a prevalence of hump‐shaped richness patterns (19 of 25 gradients), where liverwort species richness peaked at mid‐elevation and decreased towards both ends of the gradient. Against our expectation and unlike in other plant groups, in liverworts, this pattern also applies to elevational gradients at mid‐latitudes in temperate climates. Indeed, relative elevation, calculated as the percentage of the elevational range potentially inhabited by liverworts, was the most powerful predictor for the distribution of liverwort species richness. We conclude from these results that the admixture of low‐ and high‐elevation liverwort floras, in combination with steep ecological gradients, leads to a mid‐elevation floristic turnover shaping elevational patterns of liverwort diversity. Our analyses further detected significant effects of climatic variables (temperature of the warmest month, potential evapotranspiration, and precipitation of the warmest month) in explaining elevational liverwort richness patterns. This indicates that montane liverwort diversity is restricted by high temperatures and subsequent low water availability especially towards lower elevations, which presumably will lead to serious effects by temperature shifts associated with global warming.

## Graphical abstract

The investigation of elevational gradients allows to draw conclusions on factors and mechanisms determining patterns in species richness distribution. In this study, we for the first time compared worldwide data of elevational species richness patterns of liverworts and found that a hump‐shaped pattern with highest richness at the middle of the elevational extent of each gradient is the predominant pattern. Our results further give cause for serious concern about the effects of temperature shifts associated with global warming on liverwort diversity.

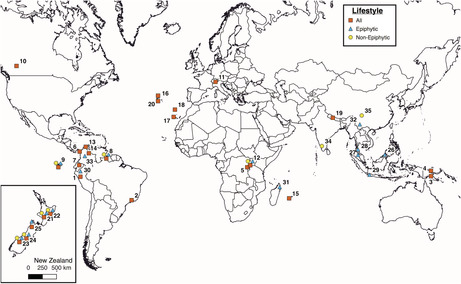

## 1 INTRODUCTION

Biodiversity is not equally distributed in space, neither latitudinally nor elevationally. The latitudinal gradient in species richness was the first ecological pattern identified and was already described by Alexander von Humboldt in the early 19th century (Hawkins, 2001). The decrease in species richness from low latitudes near the equator towards higher ones at the poles has been found to be a general pattern applying to most major taxonomic groups (e.g., foraminifera (Culver & Buzas, 2000), bats and marsupials (Lyons & Willig, 2002), vascular plants (Mutke & Barthlott, 2005)), including liverworts (Wang et al., 2017).

Quite independently of latitude, mountains are global hotspots of biodiversity (Körner, 2004). Their steep climatic gradients, together with the heterogeneity of terrain and topographical structure within short distances lead to a great variety of habitats. These promote several processes associated with speciation and maintenance of species diversity (Perrigo et al., 2020). Mountain biodiversity shows a diverse range of elevational richness patterns. In parallel to the latitudinal diversity gradient, it was long believed that species richness would mostly decline linearly with increasing elevation, until Rahbek (1995) revealed that on the majority of elevational gradients richness peaks at some intermediate point of the gradient, although other patterns (linear decreases; constant and then declining; and even increases) also occur (McCain & Grytnes, 2010; Rahbek, 2005). Nevertheless, the hump‐shaped pattern has been found to be prevalent in most organisms (Rahbek, 2005). For instance, Grytnes (2003b) found a hump‐shaped relationship between vascular plant species richness and elevation along five of seven study transects in Norway, and declining richness on the other two. For ferns on a global scale, Kessler, Kluge, et al. (2011) also found the majority of their 20 study transects to show a hump‐shaped richness pattern for ferns, but increases, decreases, and constant richness were also found.

Of course, elevation is not by itself the determining factor of species richness (Gaston, 2000). Rather, it is an indicator of a mélange of biotic and abiotic determinants, which influence species richness. Determinants to be considered are manifold (Vetaas, 2021) and include variables relevant for metabolism like the availability of energy and water such as temperature, and humidity (Kluge et al., 2017) and other environmental factors like soil properties (Liu et al., 2020; Ohdo & Takahashi, 2020; Sánchez‐González & López‐Mata, 2005), land surface area (Karger et al., 2011), past geological processes including orogeny and plate tectonics (Descombes et al., 2017; Hagen et al., 2019; Zhao & Li, 2017), phylogenetic processes such as diversification rates and phylogenetic diversity (Kluge & Kessler, 2011; Scholl & Wiens, 2016), biogeographic processes including source‐sink effects and overlaps of lowland and montane floras (Grytnes, 2003a; Kessler, Hofmann, et al., 2011). One of the major challenges in identifying these drivers of elevational richness patterns is that many potential explanatory factors covary with elevation and among each other. This is especially relevant when species richness is studied along a single transect (Kluge et al., 2006). Thus, the comparative analysis of multiple transects, which preferably exhibit different parameter peculiarities such as different climatic conditions and elevational extents, offers the opportunity to bypass this issue of covariation (Kessler, Kluge, et al., 2011; Lomolino, 2001; McCain, 2009; Vetaas et al., 2018).

Liverworts have evolved as one of the most basal groups of land plants, and comprise between 5000 and 7500 extant species (Gradstein, 2013; Von Konrat et al., 2010). Mostly being small to tiny plants, liverworts have evolved adaptations where their rhizoids allow them to grow adherent on impervious and steep surfaces, enabling them to inhabit a large variety of different habitats in addition to soil (Proctor et al., 2007) such as rocks, dead wood, tree trunks, branches, and even leaf surfaces of vascular plants (Jones & Dolan, 2012). Due to the lack of thick cuticles, liverworts desiccate quickly in the absence of liquid water and under low humidity, while suspending their metabolism. The ability to recover from desiccation (poikilohydry) is species‐specific, and its success depends on multiple conditions (e.g., duration of desiccation and humidity; León‐Vargas et al., 2006). Generally speaking, liverwort species inhabiting exposed and dry surfaces are most adapted to desiccation, followed by epiphytes of branches and the canopy. Species inhabiting tree bases and forest floor are less tolerant to drying (Franks & Bergstrom, 2000; Proctor et al., 2007), and aquatic bryophyte species are the least drought tolerant (Glime, 2017). In general, cool and humid conditions are most suitable for liverworts. Such conditions are often found at intermediate elevations of mountains (Körner et al., 2011; Rahbek, 1995).

There are various studies providing evidence for a hump‐shaped elevational richness gradient in liverworts from different parts of the world, most of them considering individual transects (Henriques et al., 2016; Tabua et al., 2017; Wolf, 1993), although some are on a regional scale (e.g., Grau et al., 2007), or compare two local data sets (Ah‐Peng et al., 2012; Iskandar et al., 2020). But divergent patterns of the elevation‐dependent change in liverwort species richness have also been described. For instance, Bruun et al. (2006) reported increasing diversity in Fennoscandia. Liverwort diversity in general has been linked to both local‐scale predictors including microhabitat variation (Ah‐Peng et al., 2007), disturbance effects (Boch et al., 2019), and bark roughness (Gradstein & Culmsee, 2010), as well as to regional‐scale factors, in particular humidity and temperature, and fog occurrence (Aranda et al., 2014; Frahm & Ohlemüller, 2001; Gehrig‐Downie et al., 2013; Henriques et al., 2016). However, a comprehensive comparative analysis of elevational gradients in species richness of liverworts is still lacking, so that no general assessment is available to date.

In this study we intended to close this knowledge gap by studying liverwort species richness on a broad diversity of mountains and mountain ranges at a global scale to address the following questions:

1. What kind of elevational richness patterns are most common for liverworts?
2. Which parameters can best explain these overall elevational richness patterns of liverworts when analyzed in combination?

## 2 MATERIALS AND METHODS

### 2.1 Data sources

**FIGURE 1:**
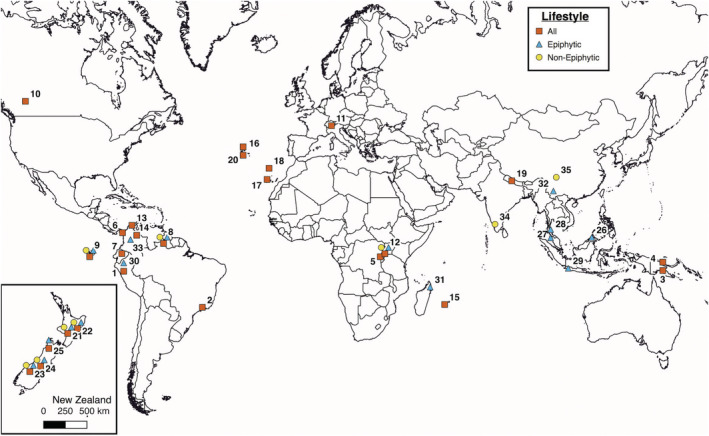
World map depicting locations of elevational gradients included in this study. Numbers correspond to Table 1.

We pooled the liverwort species richness per elevation of 50 data sets from 31 published projects/expeditions and one unpublished transect studied by us (Table 1; Appendix S1). Data obtained via various study methods were included, provided that they comprise a minimum of four elevational steps (or could be transformed into such). Furthermore, the original data must contain at least 10 taxa in total, to allow for the development of an elevational pattern in species richness. We avoided data sets in which the land surface area of mountains did not successively decline with elevation (Körner, 2000), to avoid atypical area effects caused, e.g., by high plateaus. We analyzed the species richness of 25 gradients totaling 445 taxa per elevation records from all over the world for liverwort species richness in general (i.e., without specified habitat). In addition, we conducted analyses from 16 and nine locations (163 and 90 records) for species found to inhabit epiphytic and non‐epiphytic habitats, respectively (Figure 1). Our aim was to analyze the liverwort communities at a regional rather than local scale and data on the elevational distribution of species were sometimes taken from broader, regional works. For example, elevational data on species recorded from Napo province, Ecuador, were extracted from the Ecuadorian checklist (Gradstein, 2020), even though the information in the source publication referred to the wider northern Andean region and was not restricted to the Napo gradient. The Ecuadorian data were subsequently corrected for the species with lower elevational records elsewhere than in Napo alone based on personal communication with S.R. Gradstein.

**TABLE 1:** Properties of source data included in this study.

No.	Project/expedition/publication	Life style	Study method	Location, country	Elevational sampling range (m)	Number of elevational steps	Upper gradient limit (m)
1	Bryotrop I Expedition, Schultze‐Motel and Menzel (1987)	A	Transect plots	Andes, Northeastern Peru	200–3400	17	4000
2	da Costa et al. (2015)	A	Herbarium survey	Itatiaia NP, Brazil	600–2700	10	2787
3	Koponen‐Norris Expedition Enroth (1990), Appendix S2	A	Targeted searches	Huon Peninsula, Papua New Guinea	0–3700	34	4000
4	3 transects Expedition 2nd part, this study (Appendix S1)	A	Transect plots	Mt. Wilhelm, Papua New Guinea	200–3700	8	4459
5	Bryotrop III Expedition, Fischer (1993), Appendix S2	A	Targeted searches	Kahuzi‐Biega‐NP, DRC/Nyungwe Forest, Virungas, Rwanda	850–4507	27	4507
6	Gradstein and Salazar‐Allen (1992)	A	transect plots	Darién NP, Panama	50–1150	5	1435
7	Gradstein (2020)	A	Checklist	Napo, Ecuador	300–4800	46	4800
8	Gradstein and Florschütz‐de Waard (1989)	A, E, NE	Targeted searches	Mt. Roraima, Guyana	500–2300	14	2400
9	Gradstein and Weber (1982)	A, E, NE	Herbarium survey + targeted searches	Galapagos Islands, Ecuador	10–1100	6/6/5	1300
10	Lee (1976)	A	Transect plots	Jasper NP, Canada	1067–2227	14	2694
11	Swissbryophytes (2011)–2017)	A	checklist	Switzerland	600–3000	8	3320
12	3 transects Expedition 1st part, Maul et al. (2020)	A, E, NE	Transect plots	South‐Western Uganda	680–3200	24/24/23	3645
13	van Reenen et al. (1986)	A	Transect plots	Sierra de Santa Marta, Colombia	500–4100	19	4500
14	Cacua‐Toledo et al. (2018)	A	Transect plots	Cordillera Oriental, Andes, Colombia	2400–3400	6	4020
15	Ah‐Peng et al. (2007)	A	Transect plots	Piton des Neiges, La Reunion	250–850	4	3070
16	Henriques et al. (2016)	A	Database survey	Terceira Island, Portugal	25.5–995.5	20	1021
17	Hernández‐Hernández et al. (2017)	A	Transect plots	La Palma, Spain	40–1426	8	2200
18	Boch et al. (2019)	A	Transect plots	Madeira, Portugal	91–1469	90	1861
19	Grau et al. (2007)	A	Literature survey	Central Himalaya, Nepal	100–4500	45	5200
20	Coelho et al. (2021)	A	transect plots	Pico Island, Portugal	10–2200	12	2350
21	Lindlar and Frahm (2002) + Pfeiffer (2003)	A, E, NE	Transect plots	Mt. Ruapehu, New Zealand	650–1450	5	2400
22	Lindlar and Frahm (2002) + Pfeiffer (2003)	A, E, NE	Transect plots	Urewera NP, New Zealand	600–1150	5	1392
23	Lindlar and Frahm (2002) + Pfeiffer (2003)	A, E, NE	Transect plots	Haast Pass, New Zealand	100–800	5	2000
24	Lindlar and Frahm (2002) + Pfeiffer (2003)	A, E, NE	Transect plots	Franz‐Josef Glacier, New Zealand	15–800	7/7/6	1800
25	Lindlar and Frahm (2002) + Pfeiffer (2003)	A, E	Transect plots	Karamea, New Zealand	20–1000	6	1875
26	Bryotrop II Expedition, Kürschner (1990)	E	Transect plots	Mt. Kinabalu, East Malaysia	50–3500	18	4095
27	Chantanaorrapint (2010)	E	Transect plots	Tarutao Island, Thailand	25–700	4	700
28	Chantanaorrapint and Frahm (2011) + unpublished data	E	Transect plots	Khao Luang, Khao Nan, Thailand	400–1550	8	1780
29	Iskandar et al. (2020)	E	Transect plots	Mt. Gede, Java, Indonesia	1500–2700	7	3100
30	BRYOSTRAT Project, Kürschner and Parolly (1998)	E	Transect plots	Andes, Northeastern Peru	280–3300	17	4000
31	Marline (2018)	E	Transect plots	Marojejy NP, Madagascar	250–2050	10	2132
32	Song, Ma, et al. (2015)	E	Transect plots	Mengla, Zhenyuan, Lijiang, China	800–3800	12	4000
33	Wolf (1993)	E	Transect plots	Cordillera Central, Andes, Colombia	1000–4130	15	4150
34	Alam (2011)	NE	Herbarium survey + targeted searches	Nilgiri Hills, India	1100–2600	16	2623
35	Sun et al. (2013)	NE	Transect plots	Gongga Mountains, China	2300–4220	11	4900

Abbreviations: A, all; DRC, Democratic Republic of the Congo; E, epiphytic; NE, non‐epiphytic; NP, national park.

### 2.2 Explanatory variables: elevation and climate

In order to study the relationships between species richness and elevation, as well as different climatic explanatory variables, we used the relative species richness (relRich) of each location as a response variable to give equal weight to all transects and to minimize the influence of sampling intensity and area of the individual transects. relRich was calculated by dividing the species richness of each plot or elevational belt of a given transect by the maximum species richness per plot/belt of that transect. We then transformed relRich according to Smithson and Verkuilen (2006) to fit the requirements of beta regression models.

Elevation was included both as absolute elevation (absElev) and as relative elevation (relElev) in our analyses. absElev provides information on the environmental conditions that change in relation to elevation, whereas relElev places the liverworts richness in relation to the elevational extent of the transect, which can influence richness via source‐sink effects (Kessler, Hofmann, et al., 2011), overlaps between lowland and highland floras (Grytnes, 2003a), and island effects on small mountain ranges (Kessler & Kluge, 2022). For this reason, relElev no longer reflects direct relationships with climate as does absElev but rather informs about independent biogeographical processes, which in contrast to climatic factors would also explain hump‐shaped elevational richness patterns in liverworts on mountains with short elevational extent. The quadratic term (relElev^2^) would then be expected to be a highly significant predictor for species richness. We calculated relElev of each transect by defining the elevational range potentially inhabited by liverworts, from sea level to the highest summit of each transect, unless there was a permanent snow line at highest elevations, in which case this was taken as the uppermost limit. relElev was then calculated as the percentage of the upper boundary of the respective gradient.

In the absence of exact GPS coordinates for most data points of published studies, we obtained the climate data by determining 7–12 likely coordinates in each mountain range and the respective elevations per gradient from Google Earth. These coordinates were located in the respective region of interest, as close to the original plots as it was assessable from the source publications. We then obtained Bioclim data for temperature (annual mean (Bio1), max of warmest month (Bio5), min of coldest month (Bio6)) and precipitation amount (annual (Bio12), of wettest month (Bio13), of driest month (Bio14), of warmest quarter (Bio18), and of coldest quarter (Bio19)), as well as potential evapotranspiration (PET) from CHELSA (Karger et al., 2017) for these coordinates. We selected these factors because mean parameters inform about the average conditions at a given elevation, whereas the maximum and minimum parameters approximate the likely biologically limiting factors, e.g., when the ranges of some species are determined by frost. PET was included as the maximum PET value of the year (maxPET) in our analyses. Because temperature decreases with increasing elevation (Spellman, 2013), we predicted temperatures and maxPET for our data set using linear models. The relationship between precipitation and elevation is more complex (Karger et al., 2017; McCain & Grytnes, 2010). Hence, we fitted linear or polynomial models (quadratic or cubic) for the precipitation data. We only implemented predicted precipitation variables from models with (adjusted) *R* ^2^ > .5 in our data set. Two study regions (i.e., Switzerland and Nepal) were excluded a priori because we assumed precipitation amounts to be too heterogeneous to predict with our method. For locations where detailed GPS coordinates were available (Uganda, Madagascar, Mt. Wilhelm on Papua New Guinea, Madeira), we included the according precipitation data directly, without employing models.

For the interpretation of causality, we used absElev to describe the elevational richness patterns but without assuming any direct causal influence of elevation. Rather, we used climatic factors to infer the influence of physiological limitations of the liverworts in determining their richness patterns. Finally, we used relElev as an indicator of biogeographical processes related to mountain height, independently of climate.

### 2.3 Data analyses: best predictor combinations

In the first step of analyses, we related the distribution of relRich to elevation and the climatic factors using beta regression models, allowing for both linear and unimodal relationships. To assess the goodness of model fit we used the pseudo‐*R* ^2^ ($R_{p}^{2}$) function suggested by Ferrari and Cribari‐Neto (2004) implemented in the R package betareg 3.1.‐4 (Cribari‐Neto & Zeileis, 2010). Likelihood ratio tests of nested models were conducted to decide if first or second order regression models were preferable.

In order to explain the highest possible variance of global elevational richness distribution in liverworts, we combined elevational and temperature‐related predictors with pairwise Pearson's correlation coefficients <.7 after centering the predictors (i.e., subtracting the mean). We also included quadratic terms of all explanatory variables (Model1). To test the influence of precipitation on the largest possible subset of species richness, we included the precipitation variable with the best fit in the simple regression (Model2). For the sake of comparability, we additionally regressed the reduced species richness data used for Model2 against combinations of elevational and temperature‐related predictors without precipitation (Model3).

The combined full models were ranked according to the corrected Akaike information criterion (AICc; Hurvich & Tsai, 1989), and are referred to as “best model” within ΔAICc = 2. The goodness of model fit was again assessed using $R_{p}^{2}$. Subsequently, we reduced the predictors of the best models stepwise until all predictors were significant (*p* < .001) to avoid overfitting. In order to unravel the effects from the individual versus the joint effects of predictor combinations, we conducted variance partition analyses based on partial regression: $R_{p\;\text{full model}}^{2}=R_{p\;\text{predictor}\;1}^{2}+R_{p\;\text{predictor}\;2}^{2}+R_{p\;\text{shared}}^{2}$.

All computational analyses were conducted in R 4.0.2 (R Core Team, 2017) with betareg 3.1.‐4 (Cribari‐Neto & Zeileis, 2010) to fit the beta regression models, lmtest 0.9‐38 (Zeileis & Hothorn, 2002) to conduct likelihood ratio tests of nested models, VennDiagram 1.6.0 (Chen, 2018) to draw the Venn diagrams, and the raster package 3.4‐5 (Hijmans, 2020) for the extraction of the Bioclim data.

## 3 RESULTS

### 3.1 Modeling climate data

Elevation was a significant predictor for all temperature variables and all locations (*p* < .001), with *R* ^2^ values varying between .81 and .99. The majority of the linear models relating maxPET and elevation were also significant (*p* < .001). However, significance and goodness of fit were lower in Yunnan, China (*p* < .01, *R* ^2^ = .67) and on two New Zealand transects (Karamea: *p* < .01, *R* ^2^ = .75; Haast Pass: *p* < .05, *R* ^2^ = .66).

The regressions of the precipitation variables against elevation performed very differently, so that we subsequently omitted precipitation data of locations where the *R* ^2^ values were below .5. In relation to the complete data set, which comprises 698 data points (richness records per elevational band), this resulted in reduced data sets including 473 data points for annual precipitation (Bio12), 525 for precipitation of the wettest month (Bio13), 497 for precipitation of the driest month (Bio14), 523 for precipitation of the warmest quarter (Bio18), and 481 for precipitation of the coldest quarter (Bio19). Bio13 was the precipitation variable with the least missing data for all liverworts (335 of 445 data points). Bio12 and Bio18, and Bio14 and Bio18, had the least missing values regarding epiphytic and non‐epiphytic species richness, respectively (epiphytes: 132/163, non‐epiphytes: 75/93).

### 3.2 Simple regressions

**FIGURE 2:**
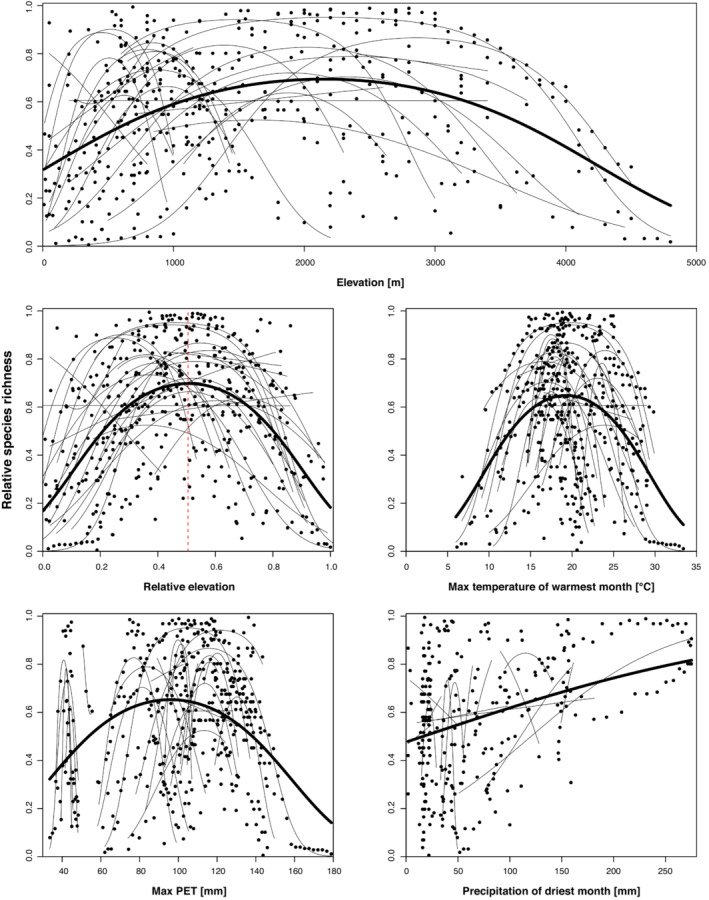
Gradients in overall liverwort relative species richness (relRich). Thin lines indicate the regression trend lines from individual locations; thick lines depict the global trend of combined relative species richness. Red dotted line marks the relative elevation of the highest global relative species richness.

Regional and global relRich distribution of all liverworts varied with absolute elevation (absElev): in 20 of 25 locations analyzed the second order polynomial of elevation explained the data best (Figure 2). The unimodal trend for species richness was least significant in Canada, as well as on both Mt. Ruapehu and the Urewera NP transects, New Zealand (*p* lrtest <.1). relRich of two locations had a significant linear relation to absolute elevation: In Darién NP in Panama, relRich increased with increasing elevation, whereas it decreased with increasing elevation on Haast Pass transect in New Zealand (*p* < .001 and .05, respectively). Further, although absElev+ absElev^2^ explained the richness data best for the transect of La Réunion, the resulting curve was U‐shaped at lower elevations and linearly increasing at the greatest part of the elevational extent. Three locations (Itatiaia NP in Brazil, Cordillera Oriental in Colombia, and Peruvian Andes) did not show a significant elevational richness pattern. The analysis of the combined regional data resulted in a unimodal relationship between relRich and absElev with an $R_{p}^{2}$ value of 0.19 (*p* < .001; Figure 2; Table 2).

**TABLE 2:** Summary of models regressing overall relative species richness.

	Coefficient estimate	Standard error	*p* value	$R_{p}^{2}$	lrtest
Intercept	−0.77	0.12	<.001		
absElev	1.5 × 10^−3^	1.5 × 10^−4^	<.001		
absElev^2^	−3.4 × 10^−7^	3.6 × 10^−8^	<.001	.19	<0.001
Intercept	−1.60	0.16	<.001		
relElev	9.65	0.76	<.001		
relElev^2^	−9.55	0.76	<.001	.28	<0.001
Intercept	−4.44	0.54	<.001		
Bio5	0.52	0.06	<.001		
Bio5^2^	−0.01	1.4 × 10^−3^	<.001	.22	<0.001
Intercept	−2.63	0.39	<.001		
maxPET	0.07	8.3 × 10^−3^	<.001		
maxPET^2^	−3.5 × 10^−4^	4.2 × 10^−5^	<.001	.16	<0.001
Intercept	−0.1	0.08			
Bio14	5.8 × 10^−3^	8.2 × 10^−4^	<.001	.17	

Abbreviations: absElev, absolute elevation; relElev, relative elevation; Bio5, max temperature of the warmest month; maxPET, maximum potential evapotranspiration; Bio14, precipitation of the driest month.

Global relRich also showed a unimodal relationship to relElev, with a maximum at almost exactly the center of the global gradient (relElev 0.51) and an $R_{p}^{2}$ value of .28 (*p* < .001). 19 of 25 individual locations showed this hump‐shaped relationship between relRich and relElev, peaking around the middle of their elevational extent, with the exceptions of the transects in Peru, Brazil, and at the Cordillera Oriental in Colombia, for which we could not find a significant relationship between relRich and relElev. The transects in Panama and on Haast Pass, New Zealand, showed a linearly increasing and decreasing trend of richness with elevation, respectively. The transect on La Réunion was U‐shaped.

The unimodal relationships between relRich and maximum temperature of the warmest month (Bio5) and maximum PET per year (maxPET) accounted for 22%, and 16% of the variance of the global data set, respectively. At the regional level, 20 of 25 locations showed a unimodal diversity–Bio5 or diversity–maxPET relationship, and the exceptions are, as above, the transects in Panama and New Zealand, which show decreasing richness with increasing temperature or maxPET (Panama) and increasing richness with increasing Bio5 or maxPET (Haast Pass, New Zealand). The transects in Brazil, Cordillera Oriental (Colombia), and Peru did not show a significant relationship between species richness and Bio5 or maxPET. The unimodal relationships between species richness and Bio5 and maxPET were only preferred with low significance to the linear relation (*p* lrtest <.1) in Jasper NP in Canada, Mt. Ruapehu, and Urewera NP transects in New Zealand.

RelRich showed a variable pattern regarding precipitation of the driest month (Bio 14), according to the fitted trend lines of the study transects. We found significant positive linear relationships in Huon Peninsula (Papua New Guinea), Darién NP (Panama), Piton des Neiges (La Reunion), and Mt. Roraima (Guyana), and negative linear relationships in one location on the North Island of New Zealand. In the Azores (Terceira and Pico Island, Portugal) and Napo (Ecuador), we recovered significant hump‐shaped relationships between relRich and Bio14. The transects in Canada and Urewera (New Zealand) showed a weak hump‐shaped relationship between relRich and Bio14 (*p* lrtest <.1). Bio14 was not significant on the remaining elevational gradients. On a global scale, species richness increased linearly with increasing precipitation, which accounted for 17% of the variance (*p* < .001; Figure 2; Table 2).

The other predictors tested were nonsignificant (Bio6), much less significant (Bio12), or accounted for less than 10% variance (Bio1, Bio13, Bio18, Bio19).

The results of analyses with relRich of global epiphytic or non‐epiphytic species richness as a dependent variable showed low explanatory power. The most meaningful predictors for global epiphytic species richness were relElev+relElev^2^ ($R_{p}^{2}$ = .07, *p* < .01) and Bio5 + Bio5^2^ ($R_{p}^{2}$ = .11, *p* < .001). Nine (56.25%) locations analyzed showed a hump‐shaped relationship between species richness and absElev, as well as relElev, respectively. Species richness was increasing with increasing elevation on the transect in Khao Luang, Thailand ($R_{p}^{2}$ = .36, *p* < .01), Yunnan, China ($R_{p}^{2}$ = .78, *p* < .001), and Urewera NP, New Zealand ($R_{p}^{2}$ = .36, *p* < .05), whereas the opposite applied to the transect on Mt. Ruapehu, New Zealand ($R_{p}^{2}$ = .7, *p* < .001). Two locations did not show a significant pattern (i.e., Peruvian Andes, and Haast Pass, New Zealand). As an exception, the transect on Tarutao Island, Thailand, shows a “reverse hump” pattern, because of a drop of observed species richness at 250 m. RelElev + relElev^2^ ($R_{p}^{2}$ = .08, *p* < .01), and Bio18 + Bio18^2^ ($R_{p}^{2}$ = .07, *p* < .05) had some validity regarding the global prediction of non‐epiphytic species richness. The relationships between species richness and elevation (relElev and absElev) were hump‐shaped on three transects (Nilgiri Hills, India ($R_{p}^{2}$ = .66, *p* < .001), Mt. Ruapehu, New Zealand ($R_{p}^{2}$ = .87, *p* < .001), Mt. Roraima, Guyana ($R_{p}^{2}$ = .57, *p* < .001)). The New Zealand Urewera ($R_{p}^{2}$ = .42, *p* < .1) and Haast Pass ($R_{p}^{2}$ = .79, *p* < .001) transects showed a decrease in non‐epiphytic species richness with increasing elevation. Four transects did not show a significant pattern (Appendix S3).

### 3.3 Best predictor combinations

**FIGURE 3:**
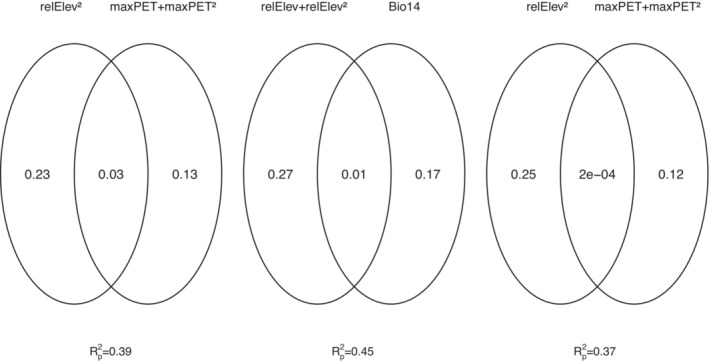
Venn diagrams show the proportions of variance in overall liverworts species richness explained by unique and joint effects of Model1, Model2, and Model3 (from left to right) after stepwise reduction of the predictor variables. *R*_p_^2^ values refer to the full model. relElev, relative elevation; Bio14, precipitation of the driest month; maxPET, maximum potential evapotranspiration.

Combining elevation and the temperature‐related variables resulted in three best models (mean $R_{p}^{2}$ = .41) in relation to relRich: relElev^2^, Bio5, Bio6, maxPET, and maxPET^2^ were part of all models (*p* < .01; Appendix S4). The combinations of relElev + Bio1, relElev + Bio5, Bio1 + Bio5, Bio1 + Bio6, and Bio1^2^ + Bio6^2^ were excluded from model selection (correlation coefficients >.7; Appendix S4). The stepwise reduction of the best models resulted in a model with slightly lower $R_{p}^{2}$ (.39), which comprised three predictors: relElev^2^ and maxPET + maxPET^2^ alone accounted for 23%, and 13% (Figure 3), respectively. The joint effect of the two variable fractions explained an additional 3%.

Regressing the reduced species richness data set against all possible combinations of relative elevation, and temperature‐related variables plus precipitation of the driest month (Bio14), resulted in 10 best models within ΔAICc ≤2 (Model2). relElev, relElev^2^, Bio5^2^ and Bio14 were constant components (*p* < .01) of all models. MaxPET^2^ and Bio6 were present in nine and six best models (Appendix S4). Further components of the best models were maxPET (five models), and Bio1^2^ and Bio6^2^ (three models each). The mean $R_{p}^{2}$‐value of the best models was .48. After stepwise reduction of the predictors, the resulting model comprised relElev, relElev^2^, and Bio14. The $R_{p}^{2}$‐value of the model slightly declined to .45, of which both terms of relative elevation accounted for the largest proportion (27%), and Bio14 for 17%. The joint effect of both fractions was negligible (1%). Regressing the same set of species richness data against elevation and temperature‐related predictors only (Model3), resulted in five best models (mean $R_{p}^{2}$ = .38) of which relElev^2^, maxPET, and maxPET^2^ were consistent components. The stepwise reduction yielded again the predictor combination relElev^2^ + maxPET + maxPET^2^ ($R_{p}^{2}$ = .37), individually contributing 25%, and 12% to the variance, respectively. There was no joint effect of the predictor fractions (<.001). The combinations relElev + Bio1, relElev + Bio5, Bio1 + Bio5, Bio1 + Bio6, and Bio1^2^ + Bio6^2^ were excluded from the Model2 and Model3 because of correlation coefficients >.7.

Combining elevation variables with climatic factors resulted in 12 and seven (including Bio14) best models with $R_{p}^{2}$‐values up to .21 (epiphytes), and seven and five (including Bio14) best models and $R_{p}^{2}$‐values up to .21 (non‐epiphytes). Stepwise reduction of the predictors was not possible to the significance level of *p* < .001 for the remaining predictors without reducing $R_{p}^{2}$‐values to a fractional amount of the more complex model, with one exception: the reduced data set of non‐epiphytic relRich (Models 2 and 3) yielded in a model containing relElev^2^ as the only predictor (*p* < .001) with a noteworthy $R_{p}^{2}$ value of .18.

## 4 DISCUSSION

This is the first study to compile a large set of elevational richness gradient studies of liverworts on a global scale and to search for general patterns. Previous studies, including some of the original publications used here, have shown a predominance of unimodal hump‐shaped patterns of liverworts species richness (e.g., Boch et al., 2019; Grau et al., 2007; Tabua et al., 2017), although other patterns have been found as well (Bruun et al., 2006; Iskandar et al., 2020; Tusiime et al., 2007). Our study confirms that, overall, the hump‐shaped pattern dominates, and that the richness peak is on average found in the middle of the elevational gradients. The prevalence of the hump‐shaped pattern in epiphytic species richness (56%), in contrast to non‐epiphytes (33%), suggests that the pattern in overall species richness is largely driven by the epiphytes.

In this study, we used a set of potential predictors to explain these elevational richness patterns. In accordance with many previous studies, we included climatic parameters related to temperature and water availability, since these can be directly related to the physiological limitations of the study group. In addition, however, we also included relative elevation as a factor. This allows a direct comparison of mountain transects of different extensions and is somewhat independent of climate. We used it as an indicator of spatial processes, such as source‐sink effects (Kessler, Hofmann, et al., 2011), overlaps between lowland and highland floras (Grytnes, 2003a), and island effects on small mountain ranges (Kessler & Kluge, 2022).

The most important result of our study is that relative elevation is shown as the most powerful single predictor of species richness. Previous studies of global elevational richness patterns of other groups of organisms, found climatic factors or absolute elevation to be dominant, although this cannot be directly compared with our study, since most of them did not calculate relative elevation. However, in a study of fern richness using a similar number of transects as in the present study, and indeed, some of the same transects, Kessler, Kluge, et al. (2011), also found a predominance of unimodal richness patterns, but in contrast to the present study, the fern transects showed a very confused pattern with low explanatory power when aligned by relative elevation. Importantly, on short transects (<2000 m), richness either increased linearly (tropics) or decreased linearly (temperate regions) with increasing elevation, as also found by Khine et al. (2019). Instead of relative elevation, the temperature was found as the best explanatory factor, and thus, Kessler, Kluge, et al. (2011) concluded that fern richness is limited at low elevations by high temperatures and high elevations by low temperatures and that richness peaks under cool (15–18°C) and moist conditions as found at mid‐elevations (1800–2000 m) along extensive tropical gradients. Considering that liverworts and ferns share a preference for cool and moist habitat conditions (Mandl et al., 2010), we here expected a similar result. Yet, this was not the case. In particular, liverwort richness showed hump‐shaped patterns even on some mountains with limited elevational extent.

In addition to relative elevation, we also found that temperature played an important role in the models combining several explanatory factors. Interestingly, the majority of the individual gradients, had a hump‐shaped relationship to Bio5, although the temperature of the richness peaks differed strongly. Temperature is a well‐known factor driving species richness patterns both because it influences ecosystem productivity and hence resource availability for organisms (Chollet et al., 2014) and because many organisms have physiological limitations directly linked to high or low temperatures (e.g., Arris & Eagleson, 1989; Breitbarth et al., 2007; Reitzel et al., 2013). Several previous studies have found temperature to be an important predictor of liverwort species richness (Chen et al., 2015; Henriques et al., 2016; Marline et al., 2020). In our study, we found maximum temperature of the warmest month (Bio5) to be the most important temperature‐related variable, outranking both mean annual temperature (Bio1) and minimum temperature of the coldest month (Bio6). This indicates that liverwort richness appears to be more strongly limited by high than low temperatures, possibly because liverworts are poikilohydric, so that they cannot control evaporative water loss and depend on constant external water supply to maintain photosynthesis (Song, Zhang, et al., 2015). Thus, although liverworts are desiccation tolerant to varying degrees (Franks & Bergstrom, 2000; León‐Vargas et al., 2006), active metabolism for growth inevitably requires available liquid water for turgescence of the cells (Proctor et al., 2007). In consequence, the growth and vitality of liverworts are reduced when they experience drought events (Song et al., 2012). Because water loss is closely linked to high temperatures, it is thus not surprising to find that these are related to liverwort richness.

The dependency of liverwort diversity on water availability is also reflected by the global trend of increasing species richness with increasing precipitation of the driest month (Bio14). For maxPET, which reflects the evaporative potential, we found a hump‐shaped richness pattern, which may be interpreted as richness being limited by low energy availability and physiological limitations under cold conditions, and by high desiccation under high‐temperature conditions.

So how can we explain the global mid‐elevation richness peak in liverworts, as relElev performs best in explaining the elevational liverwort species richness pattern but cannot be interpreted as a directly acting driver of species richness? The maximum temperature of the warmest month (Bio5) in fact correlates with elevation, but a strict climate‐based explanation as found for ferns cannot account for this pattern, since this would predict linear patterns on gradients of limited extent and a stronger predictive power of climatic factors in the analyses combining all elevational gradients. One might consider that liverwort richness is driven by climatic factors not sampled by us, such as the elevation of cloud condensation belts, which often occur at mid‐elevation on extensive tropical gradients (Bruijnzeel et al., 2011; Hemp, 2006). However, it is unlikely that this is the missing factor, since condensation belts would occur at the top of gradients to a limited extent, and because their occurrence is correlated to temperature. Indeed, although they also did not have cloud cover as a factor, Kessler, Kluge, et al. (2011) still managed to explain fern richness by climatic factors.

The most likely explanation left is that liverwort richness is indeed somehow defined by factors or processes related to the relative position along the elevation gradient. Hump‐shaped patterns of species richness have been postulated to occur when gradients are defined by strict barriers, in our case sea level and mountain top on most individual gradients (Colwell & Hurtt, 1994). In such a situation, species will overlap in the middle of the gradient, creating a hump‐shaped diversity pattern. This pattern was for a while explained via a null model, the mid‐domain effect, which assumes no biological processes beyond the random placement of species (Colwell & Hurtt, 1994). However, this model has fundamental conceptual issues and has fallen out of favor (Currie & Kerr, 2008; Hawkins et al., 2005; Kluge et al., 2006), although it may influence patterns in subtle ways (Colwell et al., 2016).

Alternatively, there are a number of biologically realistic processes leading to hump‐shaped elevational patterns. Largely, these are based on an accumulation of species at mid‐elevations that arrive from low and high elevations (Grytnes, 2003a). This overlap zone can either be caused by non‐self‐sustaining sink populations (Kessler, Hofmann, et al., 2011) or by a more permanent overlap of different floras (Grytnes, 2003a). Since the ecological conditions of mountains change within short geographical distances, liverwort community composition changes considerably with elevation (Frahm & Gradstein, 1991; Gradstein et al., 2001). The mid‐elevational richness peak may thus result from an overlap of species ranges from lower elevational zones with the ranges of species from high elevations, as previously found by Wolf (1993) for epiphytic liverworts in Colombia. This phenomenon probably shapes the global elevational gradient as well as the local gradients, where it reflects the particular relationship of the species composition with the respective environmental conditions of each gradient and marks the elevational zone of maximum species turnover. Also, the relative positions of the thermal richness peaks show that liverworts apparently do not have such a well‐defined general thermal optimum of species richness as found in ferns but are probably more dependent upon local or regional peculiarities. An additional factor to be considered in this concerns the location of the elevational gradient, as the upper natural treeline varies with latitude and relative position (continent versus island) of the respective mountain (Karger et al., 2019) and treeless mountain summits may promote a decrease in epiphytic species richness towards the upper end of the elevational gradient. We thus conclude that the most likely explanation for the prevalence of the mid‐elevation peak in liverworts is related to mountain extent, overlap of lowland and highland floras, and on some mountain‐specific peculiarities such as depressed treelines.

Besides these general trends, we also found a few exceptions along single transects that are noteworthy. Partly, these may reflect artifacts caused by differing sampling efforts. For example, da Costa et al. (2015) considered a collection deficit at 1200–2000 m in Itatiaia NP (Brazil) as a potential reason for low species richness at this elevation. Truncated sampling, where only the upper or the lower part of a gradient is sampled (McCain & Grytnes, 2010), is another possible reason for linear species richness–elevation relationships, which may apply to the Panamanian transect insofar as it is naturally truncated (mountain top at 1435 m). Further, transects in temperate regions may show linearly decreasing richness because they represent only the upper (cold) part of the full climatic gradient, as also found for ferns (Khine et al., 2019). This might also apply to the liverwort transect in Canada and two of the New Zealand transects, although other transects in New Zealand at even higher latitudes had unimodal patterns.

Finally, the models separating epiphytic and non‐epiphytic liverwort species richness contained much less data (163 records from 16 and 90 records from nine locations, respectively) than the data set for the overall species richness (where published data often did allow the separation of epiphytic and non‐epiphytic records), which probably hampered the detection of a clear global trend. Furthermore, particularly non‐epiphytic species richness of liverworts has been shown to be strongly associated with local‐scale variables such as relative humidity, vegetation structure, and exposure (Mandl et al., 2009; Maul et al., 2020), which may obscure global trends especially with limited data. We thus do not further explore the results of these analyses.

## 5 CONCLUSIONS

In this study, we for the first time compared worldwide data of elevational richness patterns of liverworts and found that a hump‐shaped pattern with the highest richness at the middle of the elevational extent of each gradient is the predominant pattern. Compared to similar studies in other plant groups, this pattern appears to be more pronounced in liverworts than in ferns (Hernández‐Rojas et al., 2021; Kessler, Kluge, et al., 2011; Khine et al., 2019), woody angiosperms (Qian, 2018; Yue & Li, 2021), seed plants (Baniya et al., 2012; Zhang et al., 2016), herbs and trees (Vetaas et al., 2019), or vascular plants in general (Grytnes, 2003b). Whether this pattern truly arises from a great floristic turnover between high‐altitude and lowland, remains to be confirmed by studies specifically assessing community structure. There is also a marked influence of climate‐related parameters on liverwort richness, particularly via high temperatures, suggesting that liverwort richness in mountains is likely to be affected by temperature shifts associated with global warming.

## AUTHOR CONTRIBUTIONS

**Karola Maul:** Conceptualization (supporting); data curation (lead); formal analysis (equal); investigation (lead); methodology (supporting); visualization (lead); writing – original draft (lead); writing – review and editing (lead). **Yu‐Mei Wei:** Resources (lead); writing – review and editing (supporting). **Eka Aditya Putri Iskandar:** Resources (supporting). **Sahut Chantanaorrapint:** Resources (supporting). **Boon‐Chuan Ho:** Resources (supporting); writing – review and editing (equal). **Dietmar Quandt:** Funding acquisition (lead); project administration (lead); resources (equal); writing – review and editing (supporting). **Michael Kessler:** Conceptualization (lead); formal analysis (equal); funding acquisition (lead); investigation (supporting); methodology (lead); supervision (lead); writing – original draft (equal); writing – review and editing (lead).

## FUNDING INFORMATION

Open Access funding enabled and organized by Projekt DEAL.

## Supporting information


Appendix S1

Appendix S2‐S4

## Data Availability

The data set of this study (i.e. species numbers, elevational distribution, and (predicted) climate data as well as the original and determined GPS coordinates for each transect) is available from the DRYAD repository at https://doi.org/10.5061/dryad.4f4qrfjg6.
