# Supplementary material for: Liverworts show a globally consistent mid‐elevation richness peak
Source: Ecol Evol. 2023 Mar 23;13(3):e9862. doi: 10.1002/ece3.9862 (PMC10034488; doi:10.1002/ece3.9862)
Supplement: Supplementary file 1 — Appendix S1 [file ECE3-13-e9862-s001.pdf]

Appendix 1. Liverwort species records and plot information of the 3 transects Expedition to Papua New Guinea. YMW, Yu-Mei Wei; MN, Martin Nebel. A total of 24 plots between 200 and 3700 m with 500 m elevational distance in between the sites were established. Eight subplots of 20 x 30 cm² each, two for each of four microhabitats (soil, rotten logs, tree trunks above 1 m, and tree branches from 2 to 4 m) were sampled in each plot, additional habitats (e.g., twigs, lianas, shrubs, small trees, tree bases etc.) were sampled outside the subplots across the entire plot, in order to capture the plot inventory as complete as possible.
Description of the study sites can be found in Leponce, M. et al. 2016. Land module of Our Planet Reviewed–Papua New Guinea: aims, methods and first taxonomical results. - In: Insects of Mount Wilhelm, Papua New Guinea. Muséum national d'Histoire naturelle, Paris, pp. 573.

**Plot**		1	2	3	4	5	6	7	8	9	10	11	12	13	14	15	16	17	18	19	20	21	22	23	24
**Latitude**		-5.8153444	-5.814967	-5.814881	-5.806996	-5.807421	-5.803817	-5.785691	-5.786214	-5.787028	-5.758982	-5.76018	-5.76096	-5.759149	-5.759938	-5.760568	-5.731967	-5.732808	-5.732247	-5.72052	-5.720889	-5.720977	-5.739838	-5.744864	-5.741027
**Longitude**		145.156281	145.157962	145.159028	145.072154	145.073585	145.070474	145.058103	145.060059	145.059741	145.186093	145.18625	145.18613	145.235891	145.234638	145.235016	145.252195	145.255726	145.256984	145.269259	145.270376	145.27155	145.329861	145.327171	145.329409
**Date of collection**		28/07/2014	28/07/2014	29/07/2014	31/07/2014	31/07/2014	31/07/2014	02/08/2014	02/08/2014	02/08/2014	05/08/2014	05/08/2014	06/08/2014	07/08/2014	07/08/2014	07/08/2014	09/08/2014	09/08/2014	09/08/2014	10/08/2014	10/08/2014	10/08/2014	11/08/2014	11/08/2014	11/08/2014
**Exposition**		SW	SW	SW	SE	SE	NE	SW	SW	NE	S	SE	SW	N	W	N	N	N	N	N	W	W	NE	-	N
**Inclination [°]**		35	10	30	15	30	25	10	10	10	20	20	40	10	10	10	40	5	5	15	35	15	10	0	10
**Altitude [m.a.s.l.]**		2700	2700	2700	3200	3200	3200	3700	3700	3700	2200	2200	2200	1700	1700	1700	700	700	700	1200	1200	1200	200	200	200
	**det.**																								
Acrobolbus ciliatus (Mitt.) Schiffn.	YMW								X																
Acrolejeunea pycnoclada (Taylor) Schiffn.	YMW																		X					X	
Acromastigum bancanum (Sande Lac.) A. Evans	YMW								X																
Acromastigum divaricatum (Nees) A. Evans ex Reimers	YMW													X						X					
Acromastigum echinatiforme (De Not.) A. Evans	YMW											X													
Acromastigum sp.	YMW	X						X																	
Anastrophyllum piligerum (Nees) Steph.	YMW			X																					
Anastrophyllum squarrosum Herzog	YMW							X	X	X															
Andrewsianthus sp.	YMW					X																			
Aneura giangena Hewson	MN					X														X	X				
Aneura kaguaensis Hewson	MN																				X				
Aneura novaguineensis Hewson	MN	X	X	X	X		X		X					X											
Aneura sp.	MN											X										X			
Balantiopsis ciliaris var. *novoguineensis* S. Hatt.	YMW	X	X								X			X	X	X				X					
Bazzania asymmetrica (Steph.) N. Kitag.	YMW			X							X														
Bazzania cf. *manillana* (Gottsche ex Steph.) S. Hatt.	YMW												X		X										
Bazzania densa (Sande Lac.) Schiffn.	YMW																				X				
Bazzania erosa (Reinw., Blume & Nees) Trevis.	YMW			X	X						X	X		X	X	X				X					
Bazzania gedeana (Steph.) Meijer	YMW													X											
Bazzania intermedia (Gottsche & Lindenb.) Trevis.	YMW										X														

For Review Only

Bazzania levieri (Steph.) N. Kitag.	YMW	X	X	X																					
Bazzania loricata (Reinw., Blume & Nees) Trevis.	YMW			X																					
Bazzania missionum (Herzog) Jovet-Ast	YMW														X										
Bazzania morokensis (Steph.) Grolle	YMW										X	X			X	X				X	X				
Bazzania pectinata (Lindenb. & Gottsche) Schiffn.	YMW										X					X									
Bazzania perfalcata N. Kitag.	YMW								X	X															
Bazzania praerupta (Reinw., Blume & Nees) Trevis.	YMW	X	X	X	X	X	X	X		X															
Bazzania scalaris Meagher	YMW										X	X													
Bazzania schusteriana N. Kitag.	YMW										X	X			X	X									
Bazzania sp.2	YMW		X																						
Bazzania sp.3	YMW										X	X	X	X											
Bazzania tridens (Reinw., Blume & Nees) Trevis.	YMW		X	X											X					X		X			
Bazzania uncigera (Reinw., Blume & Nees) Trevis.	YMW										X														
Bazzania vittata (Gottsche) Trevis.	YMW	X		X	X	X						X	X	X	X						X				
Calypogeia arguta Nees & Mont.	YMW																			X					
Calypogeia goebelii (Schiffn.) Steph.	YMW	X				X	X	X												X	X				
Caudalejeunea reniloba (Gottsche) Steph.	YMW																	X						X	X
Ceratolejeunea belangeriana (Gottsche) Steph.	YMW																X							X	
Cheilolejeunea celebensis (Steph.) Mizut.	YMW			X		X			X	X						X									
Cheilolejeunea ceylanica (Gottsche) R.M. Schust. & Kachroo	YMW	X	X	X		X	X		X					X	X	X	X	X		X	X			X	
Cheilolejeunea cf. *surrepens* (Mitt.) E.W. Jones	YMW							X																	
Cheilolejeunea cookiensis (Steph.) R.M. Schust. & Kachroo	YMW			X							X														
Cheilolejeunea falsinervis (Sande Lac.) R.M. Schust. & Kachroo	YMW													X		X									
Cheilolejeunea germanii (Besch. & Spruce) Grolle	YMW	X											X												
Cheilolejeunea gigantea (Steph.) R.M. Schust. & Kachroo	YMW							X						X											
Cheilolejeunea gradsteinii (Grolle & Piippo) W. Ye & R.L. Zhu	YMW		X												X	X									
Cheilolejeunea incisa (Gottsche) R.M. Schust. & Kachroo	YMW	X	X		X			X																	
Cheilolejeunea loriana (Steph.) W. Ye & R.L. Zhu	YMW			X	X		X		X	X						X									
Cheilolejeunea meyeniana (Nees, Lindenb. & Gottsche) R.M. Schust. & Kachroo	YMW														X										
Cheilolejeunea obtusilobula (S. Hatt.) S. Hatt.	YMW																		X						
Cheilolejeunea rigidula (Nees ex Mont.) R.M. Schust.	YMW																		X						
Cheilolejeunea sp.	YMW					X	X																		
Cheilolejeunea suprema(Grolle & Piippo) W. Ye & R.L. Zhu	YMW	X					X																		
Cheilolejeunea trapezia(Nees) Kachroo & R.M. Schust.	YMW															X			X						
Cheilolejeunea trifaria(Reinw., Blume & Nees) Mizut.	YMW			X								X													
Cheilolejeunea verrucosa Steph.	YMW																X	X					X	X	X

For Review Only

Cheilolejeunea vittata(Steph. ex G. Hoffm.) R.M. Schust. & Kachroo	YMW	X	X	X		X	X									X		X							
Cheilolejeunea xanthocarpa(Lehm. & Lindenb.) Malombe	YMW																		X						
Chiastocaulon dendroides(Nees) Carl	YMW	X	X	X	X	X	X				X		X	X	X	X	X				X	X			
Chiastocaulon theriotianum(Steph.) S.D.F. Patzak, M.A.M. Renner, Schäf.-Verw. & Heinrichs	YMW				X																				
Cololejeunea altimontana Pócs	YMW							X																	
Cololejeunea angustiflora (Steph.) Mizut.	YMW				X	X								X											
Cololejeunea appressa	YMW																	X							
Cololejeunea ciliata Pócs	YMW	X																							
Cololejeunea cordiflora Steph.	YMW																							X	
Cololejeunea hasskarliana (Lehm.) Schiffn.	YMW													X	X										
Cololejeunea inflectens (Mitt.) Benedix	YMW															X			X						
Cololejeunea metzgeriopsis (K.I. Goebel) Gradst., R. Wilson, Ilk.-Borg. & Heinrichs	YMW												X												
Cololejeunea obliqua (Nees & Mont.) Schiffn.	YMW															X									
Cololejeunea peraffinis (Schiffn.) Schiffn.	YMW	X	X	X	X						X														
Cololejeunea pseudoserrata Tixier	YMW			X							X			X	X	X									
Cololejeunea serrata (Steph.) Benedix	YMW																				X				
Cololejeunea sp.1	YMW																				X				
Cololejeunea sp.2	YMW																							X	
Cololejeunea tenella Benedix	YMW																		X					X	
Cololejeunea wightii Steph.	YMW																						X		
Colura pallida Steph.	YMW																X								
Colura tenuicornis(A. Evans) Steph.	YMW														X										
Cryptolophocolea cf. *levieri* (Schiffn.) L. Söderstr.	YMW			X																					
Cryptolophocolea ciliolata (Nees) L. Söderstr., Crand.-Stotl., Stotler & Váňa	YMW	X	X	X								X				X									
Cryptolophocolea costata (Nees) L. Söderstr.	YMW				X		X	X	X	X		X										X			
Cryptolophocolea levieri (Schiffn.) L. Söderstr.	YMW	X	X	X	X				X		X		X	X	X	X									
Cryptolophocolea sp.	YMW			X																					
Cuspidatula flaccida (Steph.) K. Feldberg, Váňa, Hentschel & Heinrichs	YMW											X	X												
Cuspidatula orbicularis (Grolle) Váňa & L. Söderstr	YMW							X	X	X															
Cylindrocolea kiaeri (Austin) Váňa	YMW			X																	X				
Denotarisia linguifolia (De Not.) Grolle	YMW										X														
Drepanolejeunea angustifolia (Mitt.) Grolle	YMW	X				X	X																		
Drepanolejeunea dactylophora (Nees, Lindenb. & Gottsche) J.B. Jack & Steph.	YMW	X	X	X			X							X	X	X				X					
Drepanolejeunea fissicornua Steph.	YMW	X	X	X			X										X								
Drepanolejeunea grandis Herzog	YMW							X																	
Drepanolejeunea nymanii Steph.	YMW	X					X																		

For Review Only

Drepanolejeunea obliqua Steph.	YMW	X																							
Drepanolejeunea serricalyx Herzog	YMW						X																		
Drepanolejeunea sp.	YMW					X																			
Drepanolejeunea ternatensis (Gottsche) Schiffn.	YMW	X	X	X										X		X			X						
Drepanolejeunea teysmanii (Gottsche) Steph.	YMW			X																					
Drepanolejeunea vesiculosa (Mitt.) Steph.	YMW		X	X				X	X	X									X						
Dumortiera hirsuta (Sw.) Nees	MN																		X						
Frullania apiculata (Reinw., Blume & Nees) Nees	YMW		X	X							X		X	X	X					X					
Frullania appendistipula S. Hatt.	YMW							X																	
Frullania appendistipula var. *spinifera* S. Hatt.	YMW											X													
Frullania armitiana Steph.	YMW							X		X															
Frullania gaudichaudii (Nees & Mont.) Nees & Mont.	YMW																X								
Frullania hasskarliana Lindenb.	YMW											X													
Frullania intermedia (Reinw., Blume & Nees) Nees	YMW																			X	X				
Frullania junghuhniana var. *tenella* (Sande Lac.) Grolle & S. Hatt.	YMW				X			X	X	X															
Frullania leeuwenii Verd.	YMW										X	X	X												
Frullania macgregorii Steph.	YMW							X	X			X			X										
Frullania nobilis Steph.	YMW							X	X	X															
Frullania nobilis var. *cochleata* (Steph.) S. Hatt.	YMW	X																							
Frullania papuana Verd.	YMW							X	X	X															
Frullania pauciramea Steph.	YMW	X			X	X	X																		
Frullania saepisdentata S. Hatt. & Streimann	YMW											X	X		X										
Frullania serrata Gottsche	YMW												X			X									
Frullania subdentata Steph.	YMW		X																						
Frullania ternatensis Gottsche	YMW					X																			
Frullania ternatensis var. *nonappendiculata* S. Hatt.	YMW						X																		
Frullania trichodes Mitt.	YMW													X											
Gottschelia schizopleura (Spruce) Grolle	YMW		X																						
Haplomitrium blumei (Nees) R.M. Schust.	YMW			X						X		X	X							X	X	X			
Harpalejeunea pinaundensis Grolle	YMW					X				X															
Harpalejeunea sp.	YMW					X			X																
Herbertus armitianus (Steph.) H.A. Mill.	YMW	X	X	X	X	X	X	X	X	X	X		X												
Herbertus circinatus (Steph.) H.A. Mill.	YMW	X			X	X																			
Herbertus pilifer Schiffn.	YMW	X			X		X																		
Herbertus ramosus (Steph.) H.A. Mill.	YMW			X					X																
Herbertus streimannii M.L. So	YMW		X																						
Heteroscyphus acutangulus (Schiffn.) Schiffn.	YMW						X		X																
Heteroscyphus argutus (Reinw., Blume & Nees) Schiffn.	YMW																X			X	X			X	
Heteroscyphus aselliformis (Reinw., Blume & Nees) Schiffn.	YMW	X	X	X		X	X				X	X	X	X	X	X									
Heteroscyphus coalitus (Hook.) Schiffn.	YMW		X	X	X	X	X				X	X		X	X	X	X	X	X	X		X			
Heteroscyphus parvulus (Schiffn.) Schiffn.	YMW			X																					

For Review Only

Heteroscyphus sarawaketanus Piippo	YMW							X	X	X															
Heteroscyphus sp. 1	YMW								X	X	X		X		X										
Heteroscyphus sp. 2	YMW							X																	
Heteroscyphus succulentus (Gottsche) Schiffn.	YMW																			X	X	X			
Heteroscyphus timppae Pipo	YMW					X		X																	
Heteroscyphus turgidus (Schiffn.) Schiffn.	YMW							X		X															
Heteroscyphus wettsteinii (Schiffn.) Schiffn.	YMW											X			X	X					X	X			
Jamesoniella flexicaulis (Nees) Schiffn.	YMW			X																					
Jensenia decipiens (Mitt.) Grolle	MN	X																							
Jubula hutchinsiae subsp. *javanica* (Steph.) Verd.	YMW	X		X	X		X																		
Kurzia pallida Piippo	YMW								X																
Leiomitra breviseta (Steph.) R.M. Schust.	YMW	X		X	X		X				X		X	X											
Lejeunea alata Gottsche	YMW	X	X	X		X	X																		
Lejeunea albscens (Steph.) Mizut.	YMW			X																					
Lejeunea anisophylla Mont.	YMW		X														X	X	X				X	X	X
Lejeunea balazsii (Pócs) R.M. Schust.	YMW	X																							
Lejeunea cf. *micholitzii* Mizut.	YMW			X													X								
Lejeunea cocoes Mitt.	YMW			X															X						
Lejeunea crenulata Mitt.	YMW													X		X									
Lejeunea dipterota (Eifrig) G.E. Lee	YMW								X																
Lejeunea discreta Lindenb.	YMW	X	X	X	X		X	X			X			X							X	X			
Lejeunea exilis (Reinw., Blume & Nees) Grolle	YMW	X		X		X	X		X				X	X		X	X			X	X			X	
Lejeunea falcata (Pócs & J. Eggers) Pócs	YMW						X																		
Lejeunea flava(Sw.) Nees	YMW														X	X			X						
Lejeunea fleischeri (Steph.) Mizut.	YMW				X	X	X	X	X	X		X			X	X									
Lejeunea kinabalensis Mizut.	YMW			X											X	X									
Lejeunea leratii (Steph.) Mizut.	YMW														X	X	X								
Lejeunea micholitzii Mizut.	YMW	X	X		X									X	X	X	X		X		X				
Lejeunea microloba Taylor	YMW														X						X				
Lejeunea mimula Hürl.	YMW		X												X	X									
Lejeunea mizutanii Grolle	YMW							X	X	X					X										
Lejeunea papilionacea Prantl	YMW					X	X														X				
Lejeunea patersonii (Steph.) Steph.	YMW		X	X										X	X	X		X			X			X	
Lejeunea reinerae Ilk.-Borg.	YMW																				X				
Lejeunea sordida (Nees) Mont.	YMW		X	X				X						X			X	X			X		X	X	X
Lejeunea sp. 1	YMW	X	X			X	X				X	X	X		X	X									
Lejeunea sp. 10	YMW													X											
Lejeunea sp. 11	YMW					X																			
Lejeunea sp. 12	YMW															X									
Lejeunea sp. 2	YMW			X							X														
Lejeunea sp. 3	YMW													X											
Lejeunea sp. 4	YMW									X															

For Review Only

Lejeunea sp. 5	YMW	X		X																					
Lejeunea sp. 6	YMW														X	X									
Lejeunea sp. 7	YMW																				X				
Lejeunea sp. 9	YMW																							X	
Lejeunea sp. nov. 1	YMW																		X						
Lejeunea sp. nov. 2	YMW												X		X						X				
Lejeunea stenodentata M.A.M. Renner & Pócs	YMW	X	X	X																					
Lejeunea tuberculosa Steph.	YMW																X								X
Lejeunea umbilicata (Nees) Nees	YMW													X			X	X		X				X	
Lejeunea utriculata (Steph.) Mizut.	YMW			X											X										
Lejeunea wightii Lindenb.	YMW					X	X									X									
Lepicolea norrisii Piipo	YMW										X	X	X		X						X				
Lepicolea rara (Steph.) Grolle	YMW	X		X	X	X	X	X	X	X					X										
Lepidolejeunea bidentula (Steph.) R.M. Schust.	YMW																	X						X	X
Lepidolejeunea graeffei (J.B. Jack & Steph.) R.M. Schust.	YMW																X	X							
Lepidolejeunea integristipula (J.B. Jack & Steph.) R.M. Schust.	YMW													X			X	X							X
Lepidolejeunea involuta (Gottsche) Grolle	YMW	X									X				X	X									
Lepidozia cladorhiza (Reinw., Blume & Nees) Nees	YMW			X	X	X					X	X	X												
Lepidozia ferdinandi-muelleri Steph.	YMW			X									X	X											
Lepidozia hasskarliana (Gottsche, Lindenb. & Nees) Steph.	YMW	X	X	X	X	X	X			X		X	X	X											
Lepidozia trichodes (Reinw., Blume & Nees) Nees	YMW		X								X	X	X	X	X						X				
Leptolejeunea epiphylla (Mitt.) Steph.	YMW																						X		
Leptolejeunea maculata (Mitt.) Schiffn.	YMW															X									
Leptolejeunea minima Herzog	YMW	X															X							X	
Leptolejeunea sp.	YMW			X																					
Leptoscyphus huonicus Piippo	YMW									X															
Lobatiriccardia sp.	MN							X	X	X			X												
Lophocolea bidentata (L.) Dumort.	YMW							X																	
Lophocolea javanica Schiffn.	YMW							X																	
Lophocolea muricata (Lehm.) Nees	YMW	X					X																		
Lophocolea sp.	YMW									X															
Lopholejeunea applanata (Reinw., Blume & Nees) Schiffn.	YMW													X	X			X			X				X
Lopholejeunea eulopha (Taylor) Schiffn.	YMW	X	X	X		X	X	X							X		X		X	X					
Lopholejeunea evansiana Verd.	YMW	X		X			X	X	X																
Lopholejeunea herzogiana Verd.	YMW														X	X									
Lopholejeunea nigricans (Lindenb.) Steph. ex Schiffn.	YMW															X									
Lopholejeunea subfusca (Nees) Schiffn.	YMW		X				X							X		X	X	X	X	X	X		X	X	X
Lopholejeunea zollingeri (Prantl) Steph. ex Schiffn.	YMW		X				X																		
Lophozia sp.	YMW																			X					
Mastigolejeunea frauenfeldii (Reichardt) Verd.	YMW	X	X																						

For Review Only

Mastigolejeunea humilis (Gottsche) Schiffn.	YMW																						X		X
Mastigolejeunea virens (Ångstr.) Steph.	YMW																							X	X
Mastigophora diclados (Brid. ex F. Weber) Nees	YMW	X	X	X	X	X	X	X	X	X	X		X		X	X				X	X				
Metalejeunea cucullata (Reinw., Blume & Nees) Grolle	YMW	X	X	X			X							X	X	X	X	X	X		X			X	
Metzgeria australis Steph.	MN			X																					
Metzgeria cf. howeana Steph.	MN								X																
Metzgeria consanguinea Schiffn.	MN				X			X													X				
Metzgeria furcata (L.) Corda	MN					X		X	X																
Metzgeria hebridensis Steph.	MN		X				X													X		X			
Metzgeria howeana Steph.	MN				X	X		X										X				X			
Metzgeria leptoneura nitida	MN			X				X						X											
Metzgeria leptoneura sp. nov.	MN			X	X		X						X		X										
Metzgeria nitida Mitt.	MN						X				X		X		X										
Metzgeria scobina Mitt.	MN																X								
Metzgeria sp.	MN											X													
Metzgeria sp. nov. prov. 1	MN	X											X	X	X		X		X	X	X				
Microlejeunea filicuspis (Steph.) Heinrichs, Schäf.-Verw., Pócs & S. Dong	YMW																							X	
Mnioloma fuscum(Lehm.) R.M. Schust.	YMW			X							X			X	X	X									
Myriocoleopsis minutissima (Sm.) R.L. Zhu, Y. Yu & Pócs	YMW							X																	
Neolepidozia wallichiana (Gottsche) Fulford & J. Taylor	YMW	X		X							X			X	X	X				X					
Odontoschisma denudatum subsp. naviculare (Steph.) Gradst., S.C. Aranda & Vanderp.	YMW							X																	
Pallavicinia ambigua (Mitt.) Steph.	MN		X		X																				
Pallavicinia levieri Schiffn.	MN				X													X	X	X					
Plagiochila abietina (Nees) Mont. et Nees	YMW										X		X		X	X									
Plagiochila amboynensis Taylor	YMW																X	X	X						
Plagiochila bantamensis (Reinw., Blume et Nees) Mont.	YMW																X	X	X	X				X	X
Plagiochila bicornuta Steph.	YMW	X			X																				
Plagiochila chauviniana Mont.	YMW																X	X	X	X					
Plagiochila frondescens (Nees) Lindenb.	YMW															X									
Plagiochila guttisquama Inoue et Grolle	YMW											X	X												
Plagiochila gymnoclada Sande Lac.	YMW	X	X	X	X	X	X		X		X		X	X	X	X				X	X	X			
Plagiochila hampeana Gottsche	YMW	X		X							X	X	X			X									
Plagiochila huerlimannii Inoue	YMW										X	X	X		X										
Plagiochila javanica (Sw.) Nees et Mont.	YMW																	X	X						
Plagiochila junghuhniana Sande Lac.	YMW																X	X	X						
Plagiochila korthalsiana Molk.	YMW																				X				
Plagiochila kurokawae Inoue	YMW				X					X															
Plagiochila kurzii Steph.	YMW																							X	X
Plagiochila metcalfii Steph.	YMW	X	X	X		X	X																		
Plagiochila monospiris Inoue et Grolle	YMW	X	X	X										X	X										

For Review Only

Plagiochila perdentata M.L.So et Grolle	YMW													X		X									
Plagiochila pulvinata Steph.	YMW							X	X																
Plagiochila renitens (Nees) Lindenb.	YMW			X																					
Plagiochila sandei Dozy ex Sande Lac.	YMW																			X	X	X			
Plagiochila sciophila Nees	YMW					X	X																		
Plagiochila singularis Schiffn.	YMW	X			X		X					X		X	X	X						X			
Plagiochila sp. 1	YMW								X																
Plagiochila sp. 2	YMW												X												
Plagiochila spinosa M.L.So	YMW								X																
Plagiochila tamiensis Steph.	YMW			X	X	X										X									
Plagiochila tecta Inoue et Grolle	YMW				X			X																	
Plagiochila trapezoidea Lindenb.	YMW			X																					
Plagiochila vernicosa Inoue et Grolle	YMW							X		X															
Plagiochilion braunianum (Nees) S.Hatt.	YMW		X	X																					
Plagiochilion oppositum (Reinw., Blume et Nees) S.Hatt.	YMW	X		X	X	X			X	X	X	X	X	X	X	X				X	X	X			
Plagiochilion pachycephalum (De Not.) Inoue	YMW													X	X										
*Plagiochilion*sp.	YMW	X																							
Pleurozia conchifolia (Hook. & Arn.) Austin	YMW		X				X	X																	
Pleurozia gigantea (F. Weber) Lindb.	YMW		X	X			X	X	X	X	X														
Plicanthus hirtellus (F. Weber) R.M. Schust.	YMW			X				X			X					X									
Podomitrium malaccense (Steph.) Campb.	MN															X					X				
Porella acutifolia (Lehm. & Lindenb.) Trevis.	YMW																	X	X	X					
Porella geheebii (Steph.) S. Hatt.	YMW		X	X	X														X						
Psiloclada clandestina Mitt.	YMW			X							X		X	X	X	X									
Ptychanthus striatus (Lehm. & Lindenb.) Nees	YMW																			X					
Radula acuta Mitt.	YMW																	X	X	X	X				
Radula amentulosa Mitt.	YMW	X	X			X	X								X										
Radula anceps Sande Lac.	YMW		X	X										X		X	X	X	X	X	X	X		X	
Radula cf. sumatrana Steph.	YMW																						X		X
Radula densifolia Castle	YMW	X	X	X	X	X	X						X	X											
Radula formosa (C.F.W. Meissn. ex Spreng.) Nees	YMW			X			X						X			X									
Radula grollei K. Yamada & Piippo	YMW						X																		
Radula hattorii K. Yamada	YMW																	X							
Radula javanica Gottsche	YMW																X	X	X	X	X		X		X
Radula koponenii K. Yamada & Piippo	YMW																							X	
Radula lacerata Steph.	YMW	X	X	X									X												
Radula minutilobula K. Yamada & Piippo	YMW	X			X		X																		
Radula mizutanii K. Yamada	YMW						X		X																
Radula multiflora Gottsche ex Schiffn.	YMW																	X						X	X
Radula norrisii K. Yamada & Piippo	YMW					X	X																		
Radula ovalilobula K. Yamada	YMW																X	X	X	X		X		X	
Radula parvitexta Steph.	YMW						X									X									

For Review Only

Radula reflexa Nees & Mont.	YMW	X															X	X			X				
Radula retroflexa Taylor	YMW													X			X		X		X			X	
Radula sharpii K. Yamada	YMW																			X					
Radula sp.	YMW												X												
Radula tjibodensis K.I. Goebel	YMW			X																					
Radula ventricosa Steph.	YMW			X								X	X	X		X	X				X	X			
Radula vrieseana Sande Lac.	YMW																X		X					X	
Riccardia agumana Hewson	MN									X															
Riccardia angustealata (Steph.) Hewson	MN				X		X	X			X														
Riccardia argento-limbata Hewson & Grolle	MN		X		X		X	X		X															
Riccardia aspera (Steph.) Grolle	MN		X								X	X	X	X	X	X									
Riccardia bliklika Hewson	MN				X																				
Riccardia demkarmana Hewson	MN	X		X	X						X		X	X	X					X		X			
Riccardia ibana Hewson	MN									X															
Riccardia kowaldiana (Steph.) Hewson	MN	X																							
Riccardia loriana (Steph.) H.A. Mill.	MN																				X				
Riccardia omkaliensis Hewson	MN						X			X															
Riccardia pengagensis Hewson	MN	X	X		X	X					X				X							X			
Riccardia pindaundensis Hewson	MN								X	X															
Riccardia pindensis Hewson	MN			X			X													X					
Riccardia sp. nov.	MN								X	X															
Riccardia womersleyana Hewson	MN	X	X	X	X	X										X									
Saccogynidium muricellum (De Not.) Grolle	YMW	X		X							X	X		X	X	X									
Saccogynidium rigidulum (Nees) Grolle	YMW	X	X																						
Scapania javanica Gottsche	YMW			X																					
Schiffneria hyalina Steph.	YMW	X		X									X												
Schiffneriolejeunea nymannii (Steph.) Gradst. & Terken	YMW	X																							
Schistochila acuminata Steph.	YMW	X	X	X							X	X				X									
Schistochila aligera (Nees & Blume) J.B. Jack & Steph.	YMW	X		X	X	X	X	X	X	X	X	X	X	X	X	X				X	X	X			
Schistochila blumei (Nees) Trevis.	YMW						X					X	X	X	X	X				X		X			
Schistochila crinita Grolle	YMW								X	X															
Schistochila ramentacea Steph.	YMW														X	X									
Schistochila rubriseta Steph.	YMW											X													
Schistochila sciurea (Nees) Schiffn.	YMW	X		X		X	X	X	X		X		X	X											
Solenostoma appressifolium (Mitt.) Váňa & D.G. Long	YMW							X																	
Solenostoma caeleste (Inoue & Váňa) Váňa, Hentschel & Heinrichs	YMW								X																
Solenostoma truncatum (Nees) R.M. Schust. ex Váňa & D.G. Long	YMW		X																						
Spruceanthus planiusculus (Mitt.) X.Q. Shi, R.L. Zhu & Gradst.	YMW																X	X	X				X	X	X
Spruceanthus pluriplicatus (Steph.) Gradst.	YMW																			X					

For Review Only

Spruceanthus polymorphus (Sande Lac.) Verd.	YMW																			X	X				
Symphyogyna similis Grolle	MN																	X							
Syzygiella securifolia (Nees ex Lindenb.) Inoue	YMW										X		X		X										
Taxilejeunea cf. nymannii Steph.	YMW						X																		
Taxilejeunea cf. stephanii Eifrig	YMW	X																							
Taxilejeunea nymannii Steph.	YMW						X																		
Taxilejeunea sp.	YMW															X									
Telaranea kogiana (Steph.) Grolle	YMW																		X						
Telaranea lawesii (Steph.) Grolle	YMW														X	X				X	X	X			
Telaranea neesii (Lindenb.) Fulford	YMW																			X					
Telaranea trisetosa (Steph.) Grolle	YMW										X	X	X	X		X									
Temnoma setigerum (Lindenb.) R.M. Schust.	YMW	X		X			X	X		X	X		X												
Thysananthus appendiculatus Steph.	YMW			X											X	X	X					X			
Thysananthus comosus Lindenb.	YMW			X																					
Thysananthus discretus Sukkharak & Gradst.	YMW	X	X	X											X	X									
Thysananthus fruticosus (Lindenb. & Gottsche) Schiffn.	YMW																X	X	X	X	X	X		X	
Thysananthus mollis Steph.	YMW	X	X	X										X						X	X				
Thysananthus retusus (Reinw., Blume & Nees) B.M. Thiers & Gradst.	YMW																X	X				X			
Thysananthus spathulistipus (Reinw., Blume & Nees) Lindenb.	YMW															X	X			X		X			
Trichocolea pluma (Reinw., Blume & Nees) Mont.	YMW	X	X	X	X		X		X	X	X	X		X	X	X	X			X	X	X			
Trichocolea rudimentaris Steph.	YMW												X							X	X	X			
Trichocolea tomentella (Ehrh.) Dumort.	YMW				X	X	X			X	X	X							X						
Tuyamaella serratistipa S. Hatt.	YMW										X			X											
Zoopsis liukiuensis Horik.	YMW	X		X			X				X					X									
Zoopsis setigera K.I. Goebel	YMW													X											
