# Supplementary material for: Liverworts show a globally consistent mid‐elevation richness peak
Source: Ecol Evol. 2023 Mar 23;13(3):e9862. doi: 10.1002/ece3.9862 (PMC10034488; doi:10.1002/ece3.9862)
Supplement: Supplementary file 2 — Appendix S2‐S4 [file ECE3-13-e9862-s002.docx]

**Appendix 2.** Additional references of source data Koponen Norris-Expedition (**3**) and Bryotrop III Expedition (**5**).

| **3** Piippo, S. 1984. Bryophyte flora of the Huon Peninsula, Papua New Guinea. III. Haplomitriaceae, Lepicoleaceae, Herbertaceae, Pseudolepicoleaceae, Trichocoleaceae, Schistochilaceae, Balantiopsaceae, Pleuroziaceae and Porellaceae (Hepaticae) Bot. Fennici 21: 21-48.  Piippo, S. 1984. Bryophyte flora of the Huon Peninsula, Papua New Guinea. VI. Lepidoziaceae subfam. Lepidozioideae, Calypogeiaceae, Adelanthaceae, Caphaloziaceae subfam. Cephalozioideae and subfam. Odontoschismatoideae and Jubulaceae (Hepaticae). Ann. Bot. Fennici 21: 309-335.  Piippo, S. 1985. Bryophyte flora of the Huon Peninsula, Papua New Guinea. X. Jackiellaceae, Scapaniaceae, Arnelliaceae and Acrobolbaceae (Hepaticae). Acta Bot. Fennica 131: 89-97.  Piippo, S. 1985. Bryophyte flora of the Huon Peninsula, Papua New Guinea. XII. Geocalycaceae (Hepaticae). Acta Bot. Fennica 131: 129-167.  Piippo, S. 1985. Bryophyte flora of the Huon Peninsula, Papua New Guinea. XIII. *Arachniopsis* and *Kurzia* (Lepidoziaceae subfam. Lepidozioideae, Hepaticae). Acta Bot. Fennica 131: 169-179.  Hattori, S. and Piippo, S. 1986. Bryophyte flora of the Huon Peninsula, Papua New Guinea. XV. *Frullania* (Frullaniaceae, Hepaticae). Acta Bot. Fennica 133: 25-58.  Grolle, R. and Piippo, S. 1986. Bryophyte flora of the Huon Peninsula, Papua New Guinea. XVI. Pallaviciniaceae (Hepaticae). Acta Bot. Fennica 133: 59-79.  Piippo, S. 1988. Bryophyte flora of the Huon Peninsula, Papua New Guinea. XXI. *Lepicolea norrisii* (Lepicoleaceae, Hepaticae). Ann. Bot. Fennici 25: 55-57.  Piippo, S. 1988. Bryophyte flora of the Huon Peninsula, Papua New Guinea. XXII. Targioniaceae, Wiesnerellaceae, Aytoniaceae and Ricciaceae (Marchantiales, Hepaticae). Ann. Bot. Fennici 25: 97-107.  Piippo, S. 1988. Bryophyte flora of the Huon Peninsula, Papua New Guinea. XXIII. Treubiaceae, Allisoniaceae and Makinoaceae (Metzgeriales, Hepaticae). Ann. Bot. Fennici 25: 159-164.  Piippo, S. and Váňa, J. 1989. Bryophyte flora of the Huon Peninsula, Papua New Guinea. XXIX. Jungermanniaceae and Gymnomitriaceae (Hepaticae). Ann. Bot. Fennici 26: 107-125.  Piippo, S. 1989. Bryophyte flora of the Huon Peninsula, Papua New Guinea. XXX. Plagiochilaceae (Hepaticae). Ann. Bot. Fennici 26: 183-236.  Váňa, J. and Piippo, S. 1989. Bryophyte flora of the Huon Peninsula, Papua New Guinea. XXXI. Cephaloziaceae subfam. Alobielloideae, Cephaloziellaceae, Antheliaceae and Lophoziaceae (Hepaticae). Ann. Bot. Fennici 26: 263-290.  Yamada, K. and Piippo, S. 1989. Bryophyte flora of the Huon Peninsula, Papua New Guinea. XXXII. *Radula* (Radulaceae, Hepaticae). Ann. Bot. Fennici 26: 349-387.  Grolle, R. and Piippo, S. 1990. Bryophyte flora of the Huon Peninsula, Papua New Guinea. XXXVII. *Leucolejeunea* (Lejeuneaceae, Hepaticae). Ann. Bot. Fennici 27: 119-129.  Piippo, S. 1991. Bryophyte flora of the Huon Peninsula, Papua New Guinea. XXXIX. *Fossombronia* (Fossombroniaceae), and *Metzgeria* (Metzgeriaceae, Hepaticae). Acta Bot. Fennica 143: 1-22.  Piippo, S. 1991. Bryophyte flora of the Huon Peninsula, Papua New Guinea. XLIX. *Targionia* (Targioniaceae, Hepaticae). Ann. Bot. Fennici 28: 273-276.  Bischler, H. and Piippo, S. 1991. Bryophyte flora of the Huon Peninsula, Papua New Guinea. L. *Marchantia* (Marchanticeae, Hepaticae). Ann. Bot. Fennici 28: 277-301.  Piippo, S. 1992. Bryophyte flora of the Huon Peninsula, Papua New Guinea. LI. Additions and corrections to the Geocalycaceae (Hepaticae). Ann. Bot. Fennici 29: 243-248.  Pócs, T., Piippo, S., and Mizutani, M. 1995. Bryophyte flora of the Huon Peninsula, Papua New Guinea. LVI. Preliminary contributions on Lejeuneaceae (Hepaticae) 2. Ann. Bot. Fennici 32: 259-268. |
| --- |
|  |
|  |

Pócs, T. and Piippo, S. 1999. Bryophyte flora of the Huon Peninsula, Papua New Guinea. LXIV. *Aphanolejeunea* (Lejeuneaceae, Hepaticae). Acta Bot. Fennica 165: 85-102.

Pócs, T., Mizutani, M., and Piippo, S. 1994. Bryophyte flora of the Huon Peninsula, Papua New Guinea. LXV. Preliminary contributions on Lejeuneaceae (Hepaticae) 1. Ann. Bot. Fennici 31: 179-190.

Gradstein, R., He, X.-L., Piippo, S., and Mizutani, M. 1999. Bryophyte flora of the Huon Peninsula, Papua New Guinea. LXVII. Lejeuneaceae subfamily Ptychanthoideae (Hepaticae). Acta Bot. Fennica 174: 1-88.

Pócs, T. and Piippo, S. 2011. Bryophyte flora of the Huon Peninsula, Papua New Guinea. LXXIV. *Cololejeunea* (Lejeuneaceae, Hepaticae). Acta Bryolichenologica Asiatica 4: 59-137.

**5** Fischer, E. 1993. Taxonomic results of the BRYOTROP-Expedition to Zaire and Rwanda 10. Trichocoleaceae, Geocalycaceae, Acrobolbaceae, Balantiopsidaceae, Lepidoziaceae (*Telaranea*, *Arachniopsis*), Calypogeiaceae, Adelanthaceae, Porellaceae, Jubulaceae, Marchantiaceae (*Dumortiera*), Polytrichaceae. Trop. Bryol. 8: 83-97.

Bischler-Causse, H. 1993. Taxonomic results of the BRYOTROP-Expedition to Zaire and Rwanda 6. Aytoniaceae, Marchantiaceae. Trop. Bryol. 8: 53-54.

Grolle, R. 1993. Taxonomic results of the BRYOTROP-Expedition to Zaire and Rwanda 9. Pallaviciniaceae, Haplomitriaceae. Trop. Bryol. 8: 75-82.

Pócs, T. 1994. Taxonomic results of the BRYOTROP-Expedition to Zaire and Rwanda 28. Lejeuneaceae, a ramicolous collection. Trop. Bryol. 9: 131-136.

Pócs, T. 1994. Taxonomic results of the BRYOTROP-Expedition to Zaire and Rwanda 27. Lepidoziaceae, II. Trop. Bryol. 9: 123-130.

Pócs, T. 1993. Taxonomic results of the BRYOTROP-Expedition to Zaire and Rwanda 12. Metzgeriaceae, Plagiochilaceae, Lejeuneaceae (the non-epiphyllous collections). Trop. Bryol. 8: 105-125.

Tixier, P. 1995. Résultats taxonomiques de l´ éxpédition BRYOTROP au Zaire et Rwanda 30. Bryophytes épiphylles (récoltes de E. Fischer). Trop. Bryol. 11: 11-76."

Váňa, J. 1993. Taxonomic results of the BRYOTROP-Expedition to Zaire and Rwanda 11. Cephaloziaceae, Cephaloziellaceae, Gymnomitriaceae, Jungermanniaceae, Lophoziaceae. Trop. Bryol. 8,:99-103."

Yamada, K. 1993. Taxonomic results of the BRYOTROP-Expedition to Zaire and Rwanda 13. Radulaceae. Trop. Bryol. 8: 127-130.

**Appendix 3.** **Simple Regressions.**

Overall species richness ~ absElev, absolute elevation [m] (left); overall species richness ~ relElev, relative elevation (right). Transect numbers refer to Table 1.

| Transect | term | estimate | std.error | z value | p.value | $\text{R}_{\text{p}}^{\text{2}}$ | Transect | term | estimate | std.error | z value | p.value | $\text{R}_{\text{p}}^{\text{2}}$ |
| --- | --- | --- | --- | --- | --- | --- | --- | --- | --- | --- | --- | --- | --- |
| 1 | Intercept | 0.43 | 0.45 | 0.95 | 0.34 | 0.00 | 1 | Intercept | 0.43 | 0.45 | 0.95 | 0.34 | 0.00 |
| 1 | absElev | 0.00 | 0.00 | -0.01 | 1.00 | 0.00 | 1 | relElev | -0.01 | 0.86 | -0.01 | 1.0 | 0.00 |
| 2 | Intercept | 0.03 | 0.92 | 0.03 | 0.98 | 0.02 | 2 | Intercept | 0.03 | 0.92 | 0.03 | 0.98 | 0.02 |
| 2 | absElev | 0.00 | 0.00 | 0.47 | 0.64 | 0.02 | 2 | relElev | 0.68 | 1.46 | 0.47 | 0.64 | 0.02 |
| 3 | Intercept | -2.93 | 0.33 | -8.93 | 0.00 | 0.74 | 3 | Intercept | -2.93 | 0.33 | -8.93 | 0.00 | 0.74 |
| 3 | absElev | 0.00 | 0.00 | 10.65 | 0.00 | 0.74 | 3 | relElev | 17.79 | 1.67 | 10.65 | 0.00 | 0.74 |
| 3 | absElev² | 0.00 | 0.00 | -10.03 | 0.00 | 0.74 | 3 | relElev² | -17.61 | 1.76 | -10.03 | 0.00 | 0.74 |
| 4 | Intercept | -1.47 | 0.53 | -2.79 | 0.01 | 0.63 | 4 | Intercept | -1.47 | 0.53 | -2.80 | 0.01 | 0.63 |
| 4 | absElev | 0.00 | 0.00 | 3.67 | 0.00 | 0.63 | 4 | relElev | 10.53 | 2.87 | 3.67 | 0.00 | 0.63 |
| 4 | absElev² | 0.00 | 0.00 | -3.17 | 0.00 | 0.63 | 4 | relElev² | -9.92 | 3.14 | -3.17 | 0.00 | 0.63 |
| 5 | Intercept | -0.75 | 0.96 | -0.79 | 0.43 | 0.44 | 5 | Intercept | -0.75 | 0.96 | -0.79 | 0.43 | 0.44 |
| 5 | absElev | 0.00 | 0.00 | 1.34 | 0.18 | 0.44 | 5 | relElev | 4.71 | 3.52 | 1.34 | 0.18 | 0.44 |
| 5 | absElev² | 0.00 | 0.00 | -2.12 | 0.03 | 0.44 | 5 | relElev² | -6.51 | 3.08 | -2.11 | 0.03 | 0.44 |
| 6 | Intercept | -0.31 | 0.30 | -1.02 | 0.31 | 0.62 | 6 | Intercept | -0.31 | 0.30 | -1.02 | 0.31 | 0.62 |
| 6 | absElev | 0.00 | 0.00 | 3.32 | 0.00 | 0.62 | 6 | relElev | 2.32 | 0.70 | 3.32 | 0.00 | 0.62 |
| 7 | Intercept | -1.44 | 0.21 | -6.99 | 0.00 | 0.92 | 7 | Intercept | -1.44 | 0.21 | -6.99 | 0.00 | 0.92 |
| 7 | absElev | 0.00 | 0.00 | 17.61 | 0.00 | 0.92 | 7 | relElev | 19.19 | 1.09 | 17.61 | 0.00 | 0.92 |
| 7 | absElev² | 0.00 | 0.00 | -19.49 | 0.00 | 0.92 | 7 | relElev² | -20.86 | 1.07 | -19.49 | 0.00 | 0.92 |
| 8 | Intercept | -3.18 | 0.97 | -3.26 | 0.00 | 0.56 | 8 | Intercept | -3.18 | 0.97 | -3.26 | 0.00 | 0.56 |
| 8 | absElev | 0.01 | 0.00 | 3.99 | 0.00 | 0.56 | 8 | relElev | 16.61 | 4.16 | 3.99 | 0.00 | 0.56 |
| 8 | absElev² | 0.00 | 0.00 | -3.90 | 0.00 | 0.56 | 8 | relElev² | -14.35 | 3.68 | -3.90 | 0.00 | 0.56 |
| 9 | Intercept | -2.41 | 0.54 | -4.48 | 0.00 | 0.85 | 9 | Intercept | -2.41 | 0.54 | -4.48 | 0.00 | 0.85 |
| 9 | absElev | 0.01 | 0.00 | 5.30 | 0.00 | 0.85 | 9 | relElev | 18.87 | 3.56 | 5.30 | 0.00 | 0.85 |
| 9 | absElev² | 0.00 | 0.00 | -5.21 | 0.00 | 0.85 | 9 | relElev² | -24.12 | 4.63 | -5.21 | 0.00 | 0.85 |
| 10 | Intercept | -6.13 | 3.20 | -1.92 | 0.06 | 0.21 | 10 | Intercept | -6.18 | 3.21 | -1.93 | 0.05 | 0.21 |
| 10 | absElev | 0.01 | 0.00 | 1.91 | 0.06 | 0.21 | 10 | relElev | 19.35 | 10.06 | 1.92 | 0.05 | 0.21 |
| 10 | absElev² | 0.00 | 0.00 | -1.83 | 0.06 | 0.21 | 10 | relElev² | -13.88 | 7.52 | -1.84 | 0.07 | 0.21 |
| 11 | Intercept | -1.17 | 0.47 | -2.50 | 0.01 | 0.96 | 11 | Intercept | -1.17 | 0.47 | -2.50 | 0.01 | 0.96 |
| 11 | absElev | 0.01 | 0.00 | 8.27 | 0.00 | 0.96 | 11 | relElev | 17.76 | 2.15 | 8.27 | 0.00 | 0.96 |
| 11 | absElev² | 0.00 | 0.00 | -10.11 | 0.00 | 0.96 | 11 | relElev² | -19.92 | 1.97 | -10.11 | 0.00 | 0.96 |
| 12 | Intercept | -2.83 | 1.17 | -2.42 | 0.02 | 0.27 | 12 | Intercept | -2.83 | 1.17 | -2.42 | 0.02 | 0.27 |
| 12 | absElev | 0.00 | 0.00 | 3.02 | 0.00 | 0.27 | 12 | relElev | 14.50 | 4.80 | 3.02 | 0.00 | 0.27 |
| 12 | absElev² | 0.00 | 0.00 | -3.10 | 0.00 | 0.27 | 12 | relElev² | -13.91 | 4.50 | -3.10 | 0.00 | 0.27 |
| 13 | Intercept | -3.97 | 0.97 | -4.07 | 0.00 | 0.46 | 13 | Intercept | -3.97 | 0.97 | -4.07 | 0.00 | 0.46 |
| 13 | absElev | 0.00 | 0.00 | 4.35 | 0.00 | 0.46 | 13 | relElev | 18.62 | 4.28 | 4.35 | 0.00 | 0.46 |
| 13 | absElev² | 0.00 | 0.00 | -4.38 | 0.00 | 0.46 | 13 | relElev² | -17.93 | 4.10 | -4.38 | 0.00 | 0.46 |
| 14 | Intercept | 2.31 | 1.70 | 1.36 | 0.17 | 0.05 | 14 | Intercept | 2.31 | 1.70 | 1.36 | 0.17 | 0.05 |
| 14 | absElev | 0.00 | 0.00 | -0.67 | 0.50 | 0.05 | 14 | relElev | -1.56 | 2.32 | -0.67 | 0.50 | 0.05 |
| 15 | Intercept | 1.21 | 0.26 | 4.60 | 0.00 | 0.98 | 15 | Intercept | -2.34 | 0.61 | -3.83 | 0.00 | 0.40 |
| 15 | absElev | 0.00 | 0.00 | -4.20 | 0.00 | 0.98 | 15 | relElev | 9.38 | 2.69 | 3.48 | 0.00 | 0.40 |
| 15 | absElev² | 0.00 | 0.00 | 6.19 | 0.00 | 0.98 | 15 | relElev² | -7.68 | 2.53 | -3.04 | 0.00 | 0.40 |
| 16 | Intercept | -2.35 | 0.61 | -3.83 | 0.00 | 0.40 | 16 | Intercept | 1.21 | 0.26 | 4.60 | 0.00 | 0.98 |
| 16 | absElev | 0.01 | 0.00 | 3.48 | 0.00 | 0.40 | 16 | relElev | -14.22 | 3.39 | -4.20 | 0.00 | 0.98 |
| 16 | absElev² | 0.00 | 0.00 | -3.04 | 0.00 | 0.40 | 16 | relElev² | 60.03 | 9.71 | 6.18 | 0.00 | 0.98 |
| 17 | Intercept | -1.92 | 0.43 | -4.47 | 0.00 | 0.81 | 17 | Intercept | -1.92 | 0.43 | -4.47 | 0.00 | 0.81 |
| 17 | absElev | 0.01 | 0.00 | 6.28 | 0.00 | 0.81 | 17 | relElev | 18.89 | 3.01 | 6.28 | 0.00 | 0.81 |
| 17 | absElev² | 0.00 | 0.00 | -6.22 | 0.00 | 0.81 | 17 | relElev² | -26.06 | 4.19 | -6.22 | 0.00 | 0.81 |
| 18 | Intercept | -3.19 | 0.53 | -6.05 | 0.00 | 0.29 | 18 | Intercept | -3.19 | 0.53 | -6.05 | 0.00 | 0.29 |
| 18 | absElev | 0.01 | 0.00 | 5.79 | 0.00 | 0.29 | 18 | relElev | 15.17 | 2.62 | 5.79 | 0.00 | 0.29 |
| 18 | absElev² | 0.00 | 0.00 | -5.01 | 0.00 | 0.29 | 18 | relElev² | -14.85 | 2.96 | -5.01 | 0.00 | 0.29 |
| 19 | Intercept | -6.91 | 0.36 | -19.39 | 0.00 | 0.89 | 19 | Intercept | -6.91 | 0.36 | -19.39 | 0.00 | 0.89 |
| 19 | absElev | 0.01 | 0.00 | 20.15 | 0.00 | 0.89 | 19 | relElev | 31.95 | 1.59 | 20.15 | 0.00 | 0.89 |
| 19 | absElev² | 0.00 | 0.00 | -19.03 | 0.00 | 0.89 | 19 | relElev² | -29.07 | 1.53 | -19.03 | 0.00 | 0.89 |
| 20 | Intercept | -0.80 | 0.64 | -1.24 | 0.21 | 0.61 | 20 | Intercept | -0.80 | 0.64 | -1.24 | 0.21 | 0.61 |
| 20 | absElev | 0.00 | 0.00 | 2.90 | 0.00 | 0.61 | 20 | relElev | 9.85 | 3.39 | 2.90 | 0.00 | 0.61 |
| 20 | absElev² | 0.00 | 0.00 | -3.63 | 0.00 | 0.61 | 20 | relElev² | -13.39 | 3.69 | -3.63 | 0.00 | 0.61 |
| 21 | Intercept | -2.59 | 2.46 | -1.05 | 0.29 | 0.78 | 21 | Intercept | -2.59 | 2.46 | -1.05 | 0.29 | 0.78 |
| 21 | absElev | 0.01 | 0.00 | 1.90 | 0.06 | 0.78 | 21 | relElev | 22.55 | 11.85 | 1.90 | 0.06 | 0.78 |
| 21 | absElev² | 0.00 | 0.00 | -2.35 | 0.02 | 0.78 | 21 | relElev² | -31.52 | 13.40 | -2.35 | 0.02 | 0.78 |
| 22 | Intercept | -3.69 | 2.65 | -1.39 | 0.16 | 0.49 | 22 | Intercept | -3.69 | 2.65 | -1.39 | 0.16 | 0.49 |
| 22 | absElev | 0.01 | 0.01 | 2.04 | 0.04 | 0.49 | 22 | relElev | 17.93 | 8.78 | 2.04 | 0.04 | 0.49 |
| 22 | absElev² | 0.00 | 0.00 | -2.14 | 0.03 | 0.49 | 22 | relElev² | -14.91 | 6.98 | -2.14 | 0.03 | 0.49 |
| 23 | Intercept | 1.40 | 0.59 | 2.39 | 0.02 | 0.56 | 23 | Intercept | 1.45 | 0.63 | 2.30 | 0.02 | 0.53 |
| 23 | absElev | 0.00 | 0.00 | -2.16 | 0.03 | 0.56 | 23 | relElev | -5.13 | 2.48 | -2.05 | 0.04 | 0.53 |
| 24 | Intercept | -0.10 | 0.30 | -0.33 | 0.74 | 0.71 | 24 | Intercept | -0.10 | 0.30 | -0.33 | 0.74 | 0.71 |
| 24 | absElev | 0.01 | 0.00 | 4.29 | 0.00 | 0.71 | 24 | relElev | 18.21 | 4.24 | 4.29 | 0.00 | 0.71 |
| 24 | absElev² | 0.00 | 0.00 | -3.95 | 0.00 | 0.71 | 24 | relElev² | -36.48 | 9.24 | -3.95 | 0.00 | 0.71 |
| 25 | Intercept | -1.01 | 0.26 | -3.89 | 0.00 | 0.92 | 25 | Intercept | -1.01 | 0.26 | -3.91 | 0.00 | 0.92 |
| 25 | absElev | 0.01 | 0.00 | 9.10 | 0.00 | 0.92 | 25 | relElev | 24.27 | 2.67 | 9.10 | 0.00 | 0.92 |
| 25 | absElev² | 0.00 | 0.00 | -9.26 | 0.00 | 0.92 | 25 | relElev² | -43.65 | 4.71 | -9.26 | 0.00 | 0.92 |

Overall species richness ~ Bio5, max temperature of the warmest month [°C] (left), and overall species richness ~ maxPET, maximum potential evapotranspiration [mm] (right). Transect numbers refer to Table 1.

| Transect | term | estimate | std.error | z value | p.value | $\text{R}_{\text{p}}^{\text{2}}$ | Transect | term | estimate | std.error | z value | p.value | $\text{R}_{\text{p}}^{\text{2}}$ |
| --- | --- | --- | --- | --- | --- | --- | --- | --- | --- | --- | --- | --- | --- |
| 1 | Intercept | 0.42 | 1.19 | 0.35 | 0.72 | 0.00 | 1 | Intercept | 0.41 | 3.04 | 0.14 | 0.89 | 0.00 |
| 1 | Bio5 | 0.00 | 0.05 | 0.01 | 1.00 | 0.00 | 1 | maxPET | 0.00 | 0.03 | 0.01 | 1.00 | 0.00 |
| 2 | Intercept | 1.41 | 2.12 | 0.66 | 0.51 | 0.02 | 2 | Intercept | 2.06 | 3.51 | 0.59 | 0.56 | 0.02 |
| 2 | Bio5 | -0.04 | 0.10 | -0.47 | 0.64 | 0.02 | 2 | maxPET | -0.01 | 0.03 | -0.47 | 0.64 | 0.02 |
| 3 | Intercept | -10.23 | 1.23 | -8.28 | 0.00 | 0.74 | 3 | Intercept | -50.99 | 5.43 | -9.40 | 0.00 | 0.74 |
| 3 | Bio5 | 1.32 | 0.14 | 9.47 | 0.00 | 0.74 | 3 | maxPET | 1.37 | 0.14 | 9.77 | 0.00 | 0.74 |
| 3 | Bio5² | -0.04 | 0.00 | -10.03 | 0.00 | 0.74 | 3 | maxPET² | -0.01 | 0.00 | -10.03 | 0.00 | 0.74 |
| 4 | Intercept | -3.58 | 1.86 | -1.98 | 0.05 | 0.63 | 4 | Intercept | -16.57 | 6.26 | -2.65 | 0.01 | 0.63 |
| 4 | Bio5 | 0.60 | 0.22 | 2.80 | 0.01 | 0.63 | 4 | maxPET | 0.43 | 0.14 | 2.97 | 0.00 | 0.63 |
| 4 | Bio5² | -0.02 | 0.01 | -3.17 | 0.00 | 0.63 | 4 | maxPET² | 0.00 | 0.00 | -3.17 | 0.00 | 0.63 |
| 5 | Intercept | -7.50 | 2.40 | -3.12 | 0.00 | 0.44 | 5 | Intercept | -21.38 | 8.07 | -2.65 | 0.01 | 0.44 |
| 5 | Bio5 | 0.63 | 0.25 | 2.56 | 0.01 | 0.44 | 5 | maxPET | 0.38 | 0.16 | 2.36 | 0.02 | 0.44 |
| 5 | Bio5² | -0.01 | 0.01 | -2.12 | 0.03 | 0.44 | 5 | maxPET² | 0.00 | 0.00 | -2.12 | 0.03 | 0.44 |
| 6 | Intercept | 7.85 | 2.24 | 3.50 | 0.00 | 0.62 | 6 | Intercept | 12.06 | 3.51 | 3.44 | 0.00 | 0.62 |
| 6 | Bio5 | -0.29 | 0.09 | -3.32 | 0.00 | 0.62 | 6 | maxPET | -0.12 | 0.04 | -3.32 | 0.00 | 0.62 |
| 7 | Intercept | -9.82 | 0.51 | -19.18 | 0.00 | 0.92 | 7 | Intercept | -33.32 | 1.67 | -19.98 | 0.00 | 0.92 |
| 7 | Bio5 | 1.29 | 0.06 | 20.34 | 0.00 | 0.92 | 7 | maxPET | 0.69 | 0.03 | 20.03 | 0.00 | 0.92 |
| 7 | Bio5² | -0.03 | 0.00 | -19.49 | 0.00 | 0.92 | 7 | maxPET² | 0.00 | 0.00 | -19.49 | 0.00 | 0.92 |
| 8 | Intercept | -40.82 | 11.01 | -3.71 | 0.00 | 0.56 | 8 | Intercept | -120.40 | 31.59 | -3.81 | 0.00 | 0.56 |
| 8 | Bio5 | 3.54 | 0.92 | 3.85 | 0.00 | 0.56 | 8 | maxPET | 2.10 | 0.54 | 3.87 | 0.00 | 0.56 |
| 8 | Bio5² | -0.07 | 0.02 | -3.90 | 0.00 | 0.56 | 8 | maxPET² | -0.01 | 0.00 | -3.90 | 0.00 | 0.56 |
| 9 | Intercept | -228.28 | 44.46 | -5.13 | 0.00 | 0.85 | 9 | Intercept | -718.09 | 138.94 | -5.17 | 0.00 | 0.85 |
| 9 | Bio5 | 19.46 | 3.76 | 5.17 | 0.00 | 0.85 | 9 | maxPET | 34.14 | 6.58 | 5.19 | 0.00 | 0.85 |
| 9 | Bio5² | -0.41 | 0.08 | -5.21 | 0.00 | 0.85 | 9 | maxPET² | -0.41 | 0.08 | -5.21 | 0.00 | 0.85 |
| 10 | Intercept | -14.44 | 8.59 | -1.68 | 0.09 | 0.21 | 10 | Intercept | -45.96 | 26.09 | -1.76 | 0.08 | 0.21 |
| 10 | Bio5 | 1.87 | 1.05 | 1.78 | 0.08 | 0.21 | 10 | maxPET | 0.85 | 0.47 | 1.81 | 0.07 | 0.21 |
| 10 | Bio5² | -0.06 | 0.03 | -1.84 | 0.07 | 0.21 | 10 | maxPET² | 0.00 | 0.00 | -1.84 | 0.07 | 0.21 |
| 11 | Intercept | -13.48 | 1.17 | -11.50 | 0.00 | 0.96 | 11 | Intercept | -39.44 | 3.54 | -11.15 | 0.00 | 0.96 |
| 11 | Bio5 | 1.64 | 0.15 | 11.20 | 0.00 | 0.96 | 11 | maxPET | 0.70 | 0.06 | 10.77 | 0.00 | 0.96 |
| 11 | Bio5² | -0.04 | 0.00 | -10.11 | 0.00 | 0.96 | 11 | maxPET² | 0.00 | 0.00 | -10.11 | 0.00 | 0.96 |
| 12 | Intercept | -22.07 | 7.32 | -3.02 | 0.00 | 0.27 | 12 | Intercept | -74.26 | 24.15 | -3.08 | 0.00 | 0.27 |
| 12 | Bio5 | 2.00 | 0.65 | 3.09 | 0.00 | 0.27 | 12 | maxPET | 1.32 | 0.43 | 3.10 | 0.00 | 0.27 |
| 12 | Bio5² | -0.04 | 0.01 | -3.10 | 0.00 | 0.27 | 12 | maxPET² | -0.01 | 0.00 | -3.10 | 0.00 | 0.27 |
| 13 | Intercept | -6.82 | 1.67 | -4.09 | 0.00 | 0.46 | 13 | Intercept | -33.83 | 7.85 | -4.31 | 0.00 | 0.46 |
| 13 | Bio5 | 0.91 | 0.21 | 4.29 | 0.00 | 0.46 | 13 | maxPET | 0.85 | 0.20 | 4.35 | 0.00 | 0.46 |
| 13 | Bio5² | -0.03 | 0.01 | -4.38 | 0.00 | 0.46 | 13 | maxPET² | -0.01 | 0.00 | -4.38 | 0.00 | 0.46 |
| 14 | Intercept | -0.02 | 1.79 | -0.01 | 0.99 | 0.05 | 14 | Intercept | -1.02 | 3.27 | -0.31 | 0.75 | 0.05 |
| 14 | Bio5 | 0.08 | 0.11 | 0.67 | 0.50 | 0.05 | 14 | maxPET | 0.02 | 0.03 | 0.67 | 0.50 | 0.05 |
| 15 | Intercept | -96.41 | 33.51 | -2.88 | 0.00 | 0.40 | 15 | Intercept | -332.05 | 112.54 | -2.95 | 0.00 | 0.40 |
| 15 | Bio5 | 9.96 | 3.37 | 2.96 | 0.00 | 0.40 | 15 | maxPET | 15.05 | 5.03 | 2.99 | 0.00 | 0.40 |
| 15 | Bio5² | -0.26 | 0.08 | -3.04 | 0.00 | 0.40 | 15 | maxPET² | -0.17 | 0.06 | -3.04 | 0.00 | 0.40 |
| 16 | Intercept | 115.78 | 17.22 | 6.72 | 0.00 | 0.98 | 16 | Intercept | 313.86 | 48.32 | 6.50 | 0.00 | 0.98 |
| 16 | Bio5 | -9.07 | 1.41 | -6.43 | 0.00 | 0.98 | 16 | maxPET | -11.48 | 1.81 | -6.33 | 0.00 | 0.98 |
| 16 | Bio5² | 0.18 | 0.03 | 6.19 | 0.00 | 0.98 | 16 | maxPET² | 0.11 | 0.02 | 6.19 | 0.00 | 0.98 |
| 17 | Intercept | -58.83 | 9.73 | -6.04 | 0.00 | 0.81 | 17 | Intercept | -212.90 | 34.64 | -6.15 | 0.00 | 0.81 |
| 17 | Bio5 | 6.17 | 1.00 | 6.16 | 0.00 | 0.81 | 17 | maxPET | 10.50 | 1.70 | 6.19 | 0.00 | 0.81 |
| 17 | Bio5² | -0.16 | 0.03 | -6.22 | 0.00 | 0.81 | 17 | maxPET² | -0.13 | 0.02 | -6.22 | 0.00 | 0.81 |
| 18 | Intercept | -47.62 | 10.44 | -4.56 | 0.00 | 0.29 | 18 | Intercept | -146.70 | 30.83 | -4.76 | 0.00 | 0.29 |
| 18 | Bio5 | 5.53 | 1.15 | 4.80 | 0.00 | 0.29 | 18 | maxPET | 2.26 | 0.46 | 4.89 | 0.00 | 0.29 |
| 18 | Bio5² | -0.16 | 0.03 | -5.01 | 0.00 | 0.29 | 18 | maxPET² | -0.01 | 0.00 | -5.01 | 0.00 | 0.29 |
| 19 | Intercept | -13.94 | 0.87 | -15.97 | 0.00 | 0.89 | 19 | Intercept | -25.55 | 1.51 | -16.93 | 0.00 | 0.89 |
| 19 | Bio5 | 1.63 | 0.09 | 17.95 | 0.00 | 0.89 | 19 | maxPET | 0.47 | 0.03 | 18.22 | 0.00 | 0.89 |
| 19 | Bio5² | -0.04 | 0.00 | -19.03 | 0.00 | 0.89 | 19 | maxPET² | 0.00 | 0.00 | -19.03 | 0.00 | 0.89 |
| 20 | Intercept | -25.79 | 6.44 | -4.00 | 0.00 | 0.61 | 20 | Intercept | -97.09 | 25.35 | -3.83 | 0.00 | 0.61 |
| 20 | Bio5 | 2.94 | 0.77 | 3.82 | 0.00 | 0.61 | 20 | maxPET | 4.60 | 1.23 | 3.73 | 0.00 | 0.61 |
| 20 | Bio5² | -0.08 | 0.02 | -3.63 | 0.00 | 0.61 | 20 | maxPET² | -0.05 | 0.01 | -3.63 | 0.00 | 0.61 |
| 21 | Intercept | -55.46 | 21.20 | -2.62 | 0.01 | 0.78 | 21 | Intercept | -190.61 | 76.19 | -2.50 | 0.01 | 0.78 |
| 21 | Bio5 | 6.36 | 2.54 | 2.50 | 0.01 | 0.78 | 21 | maxPET | 3.31 | 1.36 | 2.43 | 0.02 | 0.78 |
| 21 | Bio5² | -0.18 | 0.08 | -2.35 | 0.02 | 0.78 | 21 | maxPET² | -0.01 | 0.01 | -2.35 | 0.02 | 0.78 |
| 22 | Intercept | -70.26 | 32.83 | -2.14 | 0.03 | 0.49 | 22 | Intercept | -225.89 | 105.10 | -2.15 | 0.03 | 0.49 |
| 22 | Bio5 | 7.87 | 3.65 | 2.16 | 0.03 | 0.49 | 22 | maxPET | 3.79 | 1.76 | 2.15 | 0.03 | 0.49 |
| 22 | Bio5² | -0.22 | 0.10 | -2.14 | 0.03 | 0.49 | 22 | maxPET² | -0.02 | 0.01 | -2.14 | 0.03 | 0.49 |
| 23 | Intercept | -9.47 | 4.53 | -2.09 | 0.04 | 0.56 | 23 | Intercept | -21.95 | 10.30 | -2.13 | 0.03 | 0.56 |
| 23 | Bio5 | 0.57 | 0.26 | 2.16 | 0.03 | 0.56 | 23 | maxPET | 0.21 | 0.10 | 2.16 | 0.03 | 0.56 |
| 24 | Intercept | -143.69 | 42.52 | -3.38 | 0.00 | 0.65 | 24 | Intercept | -633.94 | 183.79 | -3.45 | 0.00 | 0.65 |
| 24 | Bio5 | 16.53 | 4.79 | 3.45 | 0.00 | 0.65 | 24 | maxPET | 12.54 | 3.61 | 3.48 | 0.00 | 0.65 |
| 24 | Bio5² | -0.47 | 0.13 | -3.50 | 0.00 | 0.65 | 24 | maxPET² | -0.06 | 0.02 | -3.50 | 0.00 | 0.65 |
| 25 | Intercept | -109.83 | 12.00 | -9.15 | 0.00 | 0.92 | 25 | Intercept | -607.52 | 65.81 | -9.23 | 0.00 | 0.92 |
| 25 | Bio5 | 12.84 | 1.39 | 9.24 | 0.00 | 0.92 | 25 | maxPET | 12.22 | 1.32 | 9.25 | 0.00 | 0.92 |
| 25 | Bio5² | -0.37 | 0.04 | -9.26 | 0.00 | 0.92 | 25 | maxPET² | -0.06 | 0.01 | -9.26 | 0.00 | 0.92 |

Overall species richness ~ Bio14, precipitation of the driest month [mm] (left), and epiphytic species richness ~ Bio5, maximum temperature of the warmest month [°C] (right). Transect numbers refer to Table 1.

| Transect | term | estimate | std.error | z value | p.value | $\text{R}_{\text{p}}^{\text{2}}$ | Transect | term | estimate | std.error | z value | p.value | $\text{R}_{\text{p}}^{\text{2}}$ |
| --- | --- | --- | --- | --- | --- | --- | --- | --- | --- | --- | --- | --- | --- |
| 1 | Intercept | 0.17 | 0.73 | 0.23 | 0.82 | 0.01 | 8 | Intercept | -29.69 | 10.07 | -2.95 | 0.00 | 0.51 |
| 1 | Bio14 | 0.00 | 0.01 | 0.38 | 0.71 | 0.01 | 8 | Temp5 | 2.67 | 0.84 | 3.17 | 0.00 | 0.51 |
| 2 | Intercept | -0.46 | 1.89 | -0.24 | 0.81 | 0.02 | 8 | Temp5² | -0.06 | 0.02 | -3.28 | 0.00 | 0.51 |
| 2 | Bio14 | 0.03 | 0.07 | 0.48 | 0.63 | 0.02 | 9 | Intercept | -238.68 | 48.21 | -4.95 | 0.00 | 0.85 |
| 3 | Intercept | -2.96 | 0.60 | -4.90 | 0.00 | 0.46 | 9 | Temp5 | 20.26 | 4.07 | 4.98 | 0.00 | 0.85 |
| 3 | Bio14 | 0.03 | 0.00 | 5.68 | 0.00 | 0.46 | 9 | Temp5² | -0.43 | 0.09 | -5.01 | 0.00 | 0.85 |
| 4 | Intercept | 0.21 | 0.76 | 0.27 | 0.79 | 0.02 | 12 | Intercept | -24.37 | 7.23 | -3.37 | 0.00 | 0.32 |
| 4 | Bio14 | 0.00 | 0.01 | 0.45 | 0.65 | 0.02 | 12 | Temp5 | 2.17 | 0.64 | 3.41 | 0.00 | 0.32 |
| 6 | Intercept | -9.73 | 4.73 | -2.06 | 0.04 | 0.42 | 12 | Temp5² | -0.05 | 0.01 | -3.38 | 0.00 | 0.32 |
| 6 | Bio14 | 0.17 | 0.08 | 2.16 | 0.03 | 0.42 | 21 | Intercept | -6.92 | 1.85 | -3.74 | 0.00 | 0.69 |
| 7 | Intercept | -5.96 | 0.74 | -8.07 | 0.00 | 0.74 | 21 | Temp5 | 0.46 | 0.11 | 4.16 | 0.00 | 0.69 |
| 7 | Bio14 | 0.08 | 0.01 | 7.84 | 0.00 | 0.74 | 22 | Intercept | 6.35 | 2.47 | 2.57 | 0.01 | 0.35 |
| 7 | Bio14² | 0.00 | 0.00 | -6.50 | 0.00 | 0.74 | 22 | Temp5 | -0.28 | 0.14 | -2.07 | 0.04 | 0.35 |
| 8 | Intercept | -4.69 | 3.13 | -1.50 | 0.13 | 0.14 | 23 | Intercept | -8.59 | 4.86 | -1.77 | 0.08 | 0.48 |
| 8 | Bio14 | 0.07 | 0.04 | 1.72 | 0.09 | 0.14 | 23 | Temp5 | 0.52 | 0.28 | -1.84 | 0.07 | 0.48 |
| 10 | Intercept | -0.69 | 1.19 | -0.58 | 0.56 | 0.03 | 24 | Intercept | -213.11 | 39.10 | -5.45 | 0.00 | 0.82 |
| 10 | Bio14 | 0.04 | 0.06 | 0.71 | 0.48 | 0.03 | 24 | Temp5 | 24.29 | 4.41 | 5.51 | 0.00 | 0.82 |
| 12 | Intercept | 1.08 | 0.72 | 1.49 | 0.14 | 0.04 | 24 | Temp5² | -0.68 | 0.12 | -5.54 | 0.00 | 0.82 |
| 12 | Bio14 | -0.02 | 0.02 | -0.99 | 0.32 | 0.04 | 25 | Intercept | -106.42 | 12.87 | -8.27 | 0.00 | 0.91 |
| 16 | Intercept | -39.33 | 8.38 | -4.69 | 0.00 | 0.58 | 25 | Temp5 | 12.43 | 1.49 | 8.34 | 0.00 | 0.91 |
| 16 | Bio14 | 1.72 | 0.38 | 4.55 | 0.00 | 0.58 | 25 | Temp5² | -0.36 | 0.04 | -8.36 | 0.00 | 0.91 |
| 16 | Bio14² | -0.02 | 0.00 | -4.42 | 0.00 | 0.58 | 26 | Intercept | -9.67 | 2.57 | -3.77 | 0.00 | 0.35 |
| 15 | Intercept | -14.64 | 5.39 | -2.72 | 0.01 | 0.68 | 26 | Temp5 | 1.10 | 0.28 | 4.01 | 0.00 | 0.35 |
| 15 | Bio14 | 0.10 | 0.03 | 2.87 | 0.00 | 0.68 | 26 | Temp5² | -0.03 | 0.01 | -4.22 | 0.00 | 0.35 |
| 18 | Intercept | -0.15 | 0.45 | -0.33 | 0.74 | 0.00 | 27 | Intercept | 546.25 | 238.11 | 2.29 | 0.02 | 0.58 |
| 18 | Bio14 | 0.01 | 0.02 | 0.58 | 0.56 | 0.00 | 27 | Temp5 | -38.57 | 16.92 | -2.28 | 0.02 | 0.58 |
| 20 | Intercept | -69.63 | 20.56 | -3.39 | 0.00 | 0.61 | 27 | Temp5² | 0.68 | 0.30 | 2.27 | 0.02 | 0.58 |
| 20 | Bio14 | 3.56 | 1.01 | 3.51 | 0.00 | 0.61 | 28 | Intercept | 7.89 | 2.79 | 2.83 | 0.00 | 0.36 |
| 20 | Bio14² | -0.04 | 0.01 | -3.63 | 0.00 | 0.61 | 28 | Temp5 | -0.28 | 0.11 | -2.62 | 0.01 | 0.36 |
| 21 | Intercept | 6.33 | 1.84 | 3.44 | 0.00 | 0.56 | 29 | Intercept | -43.33 | 9.61 | -4.51 | 0.00 | 0.66 |
| 21 | Bio14 | -0.05 | 0.02 | -3.07 | 0.00 | 0.56 | 29 | Temp5 | 4.95 | 1.05 | 4.71 | 0.00 | 0.66 |
| 22 | Intercept | 2.35 | 1.30 | 1.81 | 0.07 | 0.07 | 29 | Temp5² | -0.13 | 0.03 | -4.74 | 0.00 | 0.66 |
| 22 | Bio14 | -0.01 | 0.01 | -0.75 | 0.45 | 0.07 | 30 | Intercept | -1.37 | 1.22 | -1.12 | 0.26 | 0.07 |
|  | | | | | | | 30 | Temp5 | 0.07 | 0.05 | 1.20 | 0.23 | 0.07 |
|  | | | | | | | 31 | Intercept | -44.34 | 14.63 | -3.03 | 0.00 | 0.48 |
|  |  |  |  |  |  |  | 31 | Temp5 | 3.91 | 1.28 | 3.06 | 0.00 | 0.48 |
|  |  |  |  |  |  |  | 31 | Temp5² | -0.08 | 0.03 | -3.07 | 0.00 | 0.48 |
|  |  |  |  |  |  |  | 32 | Intercept | 8.30 | 1.03 | 8.08 | 0.00 | 0.78 |
|  |  |  |  |  |  |  | 32 | Temp5 | -0.35 | 0.04 | -7.78 | 0.00 | 0.78 |
|  |  |  |  |  |  |  | 33 | Intercept | -10.68 | 2.19 | -4.87 | 0.00 | 0.68 |
|  |  |  |  |  |  |  | 33 | Temp5 | 1.38 | 0.25 | 5.44 | 0.00 | 0.68 |
|  |  |  |  |  |  |  | 33 | Temp5² | -0.04 | 0.01 | -5.67 | 0.00 | 0.68 |
|  |  |  |  |  |  |  | epiphytic  species richness  pooled | Intercept | -4.71 | 1.41 | -3.33 | 0.00 | 0.10 |
|  |  |  |  |  |  |  | epiphytic  species richness  pooled | Temp5 | 0.54 | 0.14 | 3.93 | 0.00 | 0.10 |
|  |  |  |  |  |  |  | epiphytic  species richness  pooled | Temp5² | -0.01 | 0.00 | -4.14 | 0.00 | 0.10 |

Epiphytic species richness ~ relElev, relative elevation (left); epiphytic species richness ~ absElev, absolute elevation [m] (right). Transect numbers refer to Table 1.

| Transect | term | estimate | std.error | z value | p.value | $\text{R}_{\text{p}}^{\text{2}}$ | Transect | term | estimate | std.error | z value | p.value | $\text{R}_{\text{p}}^{\text{2}}$ |
| --- | --- | --- | --- | --- | --- | --- | --- | --- | --- | --- | --- | --- | --- |
| 8 | Intercept | -2.39 | 0.89 | -2.68 | 0.01 | 0.51 | 8 | Intercept | -2.39 | 0.89 | -2.68 | 0.01 | 0.51 |
| 8 | relElev | 13.49 | 3.82 | 3.53 | 0.00 | 0.51 | 8 | absElev | 0.01 | 0.00 | 3.53 | 0.00 | 0.51 |
| 8 | relElev² | -11.04 | 3.37 | -3.28 | 0.00 | 0.51 | 8 | absElev² | 0.00 | 0.00 | -3.28 | 0.00 | 0.51 |
| 9 | Intercept | -2.38 | 0.56 | -4.25 | 0.00 | 0.85 | 9 | Intercept | -2.38 | 0.56 | -4.25 | 0.00 | 0.85 |
| 9 | relElev | 18.99 | 3.76 | 5.05 | 0.00 | 0.85 | 9 | absElev | 0.01 | 0.00 | 5.05 | 0.00 | 0.85 |
| 9 | relElev² | -25.01 | 4.99 | -5.01 | 0.00 | 0.85 | 9 | absElev² | 0.00 | 0.00 | -5.01 | 0.00 | 0.85 |
| 12 | Intercept | -2.83 | 1.14 | -2.48 | 0.01 | 0.32 | 12 | Intercept | -2.83 | 1.14 | -2.48 | 0.01 | 0.32 |
| 12 | relElev | 15.09 | 4.70 | 3.21 | 0.00 | 0.32 | 12 | absElev | 0.00 | 0.00 | 3.21 | 0.00 | 0.32 |
| 12 | relElev² | -14.95 | 4.42 | -3.38 | 0.00 | 0.32 | 12 | absElev² | 0.00 | 0.00 | -3.38 | 0.00 | 0.32 |
| 21 | Intercept | 3.56 | 0.70 | 5.08 | 0.00 | 0.69 | 21 | Intercept | 3.56 | 0.70 | 5.08 | 0.00 | 0.69 |
| 21 | relElev | -6.16 | 1.48 | -4.16 | 0.00 | 0.69 | 21 | absElev | 0.00 | 0.00 | -4.16 | 0.00 | 0.69 |
| 22 | Intercept | -0.21 | 0.72 | -0.28 | 0.78 | 0.35 | 22 | Intercept | -0.21 | 0.72 | -0.28 | 0.78 | 0.35 |
| 22 | relElev | 2.34 | 1.13 | 2.07 | 0.04 | 0.35 | 22 | absElev | 0.00 | 0.00 | 2.07 | 0.04 | 0.35 |
| 23 | Intercept | 1.33 | 0.63 | 2.12 | 0.03 | 0.48 | 23 | Intercept | 1.33 | 0.63 | 2.12 | 0.03 | 0.48 |
| 23 | relElev | -4.60 | 2.50 | -1.84 | 0.07 | 0.48 | 23 | absElev | 0.00 | 0.00 | -1.84 | 0.07 | 0.48 |
| 24 | Intercept | -0.38 | 0.29 | -1.32 | 0.19 | 0.82 | 24 | Intercept | -0.38 | 0.29 | -1.32 | 0.19 | 0.82 |
| 24 | relElev | 24.33 | 4.29 | 5.67 | 0.00 | 0.82 | 24 | absElev | 0.01 | 0.00 | 5.67 | 0.00 | 0.82 |
| 24 | relElev² | -51.43 | 9.29 | -5.54 | 0.00 | 0.82 | 24 | absElev² | 0.00 | 0.00 | -5.54 | 0.00 | 0.82 |
| 25 | Intercept | -1.05 | 0.28 | -3.71 | 0.00 | 0.91 | 25 | Intercept | -1.05 | 0.28 | -3.71 | 0.00 | 0.91 |
| 25 | relElev | 23.49 | 2.86 | 8.21 | 0.00 | 0.91 | 25 | absElev | 0.01 | 0.00 | 8.21 | 0.00 | 0.91 |
| 25 | relElev² | -42.26 | 5.05 | -8.36 | 0.00 | 0.91 | 25 | absElev² | 0.00 | 0.00 | -8.36 | 0.00 | 0.91 |
| 26 | Intercept | -3.24 | 0.72 | -4.49 | 0.00 | 0.35 | 26 | Intercept | -3.24 | 0.72 | -4.49 | 0.00 | 0.35 |
| 26 | relElev | 15.17 | 3.40 | 4.46 | 0.00 | 0.35 | 26 | absElev | 0.00 | 0.00 | 4.46 | 0.00 | 0.35 |
| 26 | relElev² | -14.85 | 3.52 | -4.22 | 0.00 | 0.35 | 26 | absElev² | 0.00 | 0.00 | -4.22 | 0.00 | 0.35 |
| 27 | Intercept | 1.67 | 0.67 | 2.50 | 0.01 | 0.58 | 27 | Intercept | 1.67 | 0.67 | 2.50 | 0.01 | 0.58 |
| 27 | relElev | -6.17 | 3.11 | -1.98 | 0.05 | 0.58 | 27 | absElev | -0.01 | 0.00 | -1.98 | 0.05 | 0.58 |
| 27 | relElev² | 6.90 | 3.04 | 2.27 | 0.02 | 0.58 | 27 | absElev² | 0.00 | 0.00 | 2.27 | 0.02 | 0.58 |
| 28 | Intercept | -0.84 | 0.60 | -1.41 | 0.16 | 0.36 | 28 | Intercept | -0.84 | 0.60 | -1.41 | 0.16 | 0.36 |
| 28 | relElev | 2.66 | 1.02 | 2.62 | 0.01 | 0.36 | 28 | absElev | 0.00 | 0.00 | 2.62 | 0.01 | 0.36 |
| 29 | Intercept | -16.79 | 3.81 | -4.41 | 0.00 | 0.66 | 29 | Intercept | -16.79 | 3.81 | -4.41 | 0.00 | 0.66 |
| 29 | relElev | 56.02 | 11.72 | 4.78 | 0.00 | 0.66 | 29 | absElev | 0.02 | 0.00 | 4.78 | 0.00 | 0.66 |
| 29 | relElev² | -40.97 | 8.64 | -4.74 | 0.00 | 0.66 | 29 | absElev² | 0.00 | 0.00 | -4.74 | 0.00 | 0.66 |
| 30 | Intercept | 0.56 | 0.46 | 1.22 | 0.22 | 0.07 | 30 | Intercept | 0.56 | 0.46 | 1.22 | 0.22 | 0.07 |
| 30 | relElev | -1.06 | 0.88 | -1.20 | 0.23 | 0.07 | 30 | absElev | 0.00 | 0.00 | -1.20 | 0.23 | 0.07 |
| 31 | Intercept | -1.91 | 0.86 | -2.23 | 0.03 | 0.48 | 31 | Intercept | -1.91 | 0.86 | -2.23 | 0.03 | 0.48 |
| 31 | relElev | 11.06 | 3.69 | 3.00 | 0.00 | 0.48 | 31 | absElev | 0.01 | 0.00 | 3.00 | 0.00 | 0.48 |
| 31 | relElev² | -10.27 | 3.35 | -3.07 | 0.00 | 0.48 | 31 | absElev² | 0.00 | 0.00 | -3.07 | 0.00 | 0.48 |
| 32 | Intercept | -2.34 | 0.38 | -6.14 | 0.00 | 0.78 | 32 | Intercept | -2.34 | 0.38 | -6.14 | 0.00 | 0.78 |
| 32 | relElev | 5.46 | 0.70 | 7.78 | 0.00 | 0.78 | 32 | absElev | 0.00 | 0.00 | 7.78 | 0.00 | 0.78 |
| 33 | Intercept | -6.92 | 1.27 | -5.43 | 0.00 | 0.68 | 33 | Intercept | -6.92 | 1.27 | -5.43 | 0.00 | 0.68 |
| 33 | relElev | 26.20 | 4.49 | 5.83 | 0.00 | 0.68 | 33 | absElev | 0.01 | 0.00 | 5.83 | 0.00 | 0.68 |
| 33 | relElev² | -20.42 | 3.60 | -5.67 | 0.00 | 0.68 | 33 | absElev² | 0.00 | 0.00 | -5.67 | 0.00 | 0.68 |
| epiphytic  species richness  pooled | Intercept | -0.39 | 0.25 | -1.54 | 0.12 | 0.07 | epiphytic  species richness  pooled | Intercept | 0.05 | 0.20 | 0.26 | 0.79 | 0.03 |
| epiphytic  species richness  pooled | relElev | 3.54 | 1.13 | 3.12 | 0.00 | 0.07 | epiphytic  species richness  pooled | absElev | 0.00 | 0.00 | 2.14 | 0.03 | 0.03 |
| epiphytic  species richness  pooled | relElev² | -3.00 | 1.12 | -2.67 | 0.01 | 0.07 | epiphytic  species richness  pooled | absElev² | 0.00 | 0.00 | -2.07 | 0.04 | 0.03 |

Non-epiphytic species richness ~ absElev, absolute elevation [m]; non-epiphytic species richness ~ Bio18, precipitation of the warmest month [mm] (left); non-epiphytic species richness ~ relElev, relative elevation (right). Transect numbers refer to

Table 1.

| Transect | term | estimate | std.error | z value | p.value | $\text{R}_{\text{p}}^{\text{2}}$ | Transect | term | estimate | std.error | z value | p.value | $\text{R}_{\text{p}}^{\text{2}}$ |
| --- | --- | --- | --- | --- | --- | --- | --- | --- | --- | --- | --- | --- | --- |
| 8 | Intercept | -3.29 | 1.07 | -3.07 | 0.00 | 0.57 | 8 | Intercept | -3.29 | 1.07 | -3.07 | 0.00 | 0.57 |
| 8 | absElev | 0.01 | 0.00 | 4.04 | 0.00 | 0.57 | 8 | relElev | 18.74 | 4.64 | 4.04 | 0.00 | 0.57 |
| 8 | absElev² | 0.00 | 0.00 | -4.13 | 0.00 | 0.57 | 8 | relElev² | -17.16 | 4.15 | -4.14 | 0.00 | 0.57 |
| 9 | Intercept | -0.84 | 0.87 | -0.97 | 0.34 | 0.20 | 9 | Intercept | -0.84 | 0.87 | -0.97 | 0.34 | 0.20 |
| 9 | absElev | 0.00 | 0.00 | 1.02 | 0.31 | 0.20 | 9 | relElev | 1.91 | 1.88 | 1.02 | 0.31 | 0.20 |
| 12 | Intercept | -0.74 | 0.64 | -1.17 | 0.24 | 0.06 | 12 | Intercept | -0.74 | 0.64 | -1.17 | 0.24 | 0.06 |
| 12 | absElev | 0.00 | 0.00 | 1.11 | 0.27 | 0.06 | 12 | relElev | 1.24 | 1.12 | 1.11 | 0.27 | 0.06 |
| 21 | Intercept | -15.44 | 2.77 | -5.57 | 0.00 | 0.87 | 21 | Intercept | -15.44 | 2.77 | -5.57 | 0.00 | 0.87 |
| 21 | absElev | 0.03 | 0.01 | 5.71 | 0.00 | 0.87 | 21 | relElev | 76.62 | 13.43 | 5.71 | 0.00 | 0.87 |
| 21 | absElev² | 0.00 | 0.00 | -5.69 | 0.00 | 0.87 | 21 | relElev² | -86.45 | 15.20 | -5.69 | 0.00 | 0.87 |
| 22 | Intercept | 3.71 | 1.93 | 1.93 | 0.05 | 0.42 | 22 | Intercept | 3.71 | 1.93 | 1.93 | 0.05 | 0.42 |
| 22 | absElev | 0.00 | 0.00 | -1.92 | 0.05 | 0.42 | 22 | relElev | -5.57 | 2.90 | -1.92 | 0.05 | 0.42 |
| 23 | Intercept | 1.41 | 0.41 | 3.43 | 0.00 | 0.79 | 23 | Intercept | 1.41 | 0.41 | 3.43 | 0.00 | 0.79 |
| 23 | absElev | 0.00 | 0.00 | -4.12 | 0.00 | 0.79 | 23 | relElev | -7.22 | 1.75 | -4.12 | 0.00 | 0.79 |
| 24 | Intercept | -0.01 | 0.54 | -0.03 | 0.98 | 0.33 | 24 | Intercept | -0.01 | 0.54 | -0.03 | 0.98 | 0.33 |
| 24 | absElev | 0.00 | 0.00 | 1.38 | 0.17 | 0.33 | 24 | relElev | 3.36 | 2.44 | 1.38 | 0.17 | 0.33 |
| 34 | Intercept | -20.26 | 3.49 | -5.80 | 0.00 | 0.66 | 34 | Intercept | -20.26 | 3.49 | -5.80 | 0.00 | 0.66 |
| 34 | absElev | 0.03 | 0.00 | 6.22 | 0.00 | 0.66 | 34 | relElev | 66.49 | 10.69 | 6.22 | 0.00 | 0.66 |
| 34 | absElev² | 0.00 | 0.00 | -6.41 | 0.00 | 0.66 | 34 | relElev² | -50.75 | 7.92 | -6.41 | 0.00 | 0.66 |
| 35 | Intercept | 1.15 | 1.65 | 0.70 | 0.49 | 0.04 | 35 | Intercept | 1.15 | 1.65 | 0.70 | 0.49 | 0.04 |
| 35 | absElev | 0.00 | 0.00 | -0.67 | 0.51 | 0.04 | 35 | relElev | -1.56 | 2.34 | -0.67 | 0.51 | 0.04 |
| non-epiphytic  species richness  pooled | Intercept | 0.29 | 0.20 | 1.44 | 0.15 | 0.00 | non-epiphytic  species richness  pooled | Intercept | -0.66 | 0.40 | -1.65 | 0.10 | 0.08 |
| non-epiphytic  species richness  pooled | absElev | 0.00 | 0.00 | -0.59 | 0.56 | 0.00 | non-epiphytic  species richness  pooled | relElev | 4.59 | 1.73 | 2.66 | 0.01 | 0.08 |
| non-epiphytic  species richness  pooled | Intercept | -1.66 | 0.85 | -1.96 | <0.05 | 0.07 | non-epiphytic  species richness  pooled | relElev² | -4.67 | 1.69 | -2.75 | 0.01 | 0.08 |
| non-epiphytic  species richness  pooled | Bio18 | 0.01 | 0.01 | 2.12 | 0.03 | 0.07 |  |  |  |  |  |  |  |
| non-epiphytic  species richness  pooled | Bio18² | 0.00 | 0.00 | -1.98 | <0.05 | 0.07 |  |  |  |  |  |  |  |

**Appendix 4.** Correlation tables and best models for overall species richness within ΔAICc ≤ 2. absElev, absolute elevation [m]; relElev, relative elevation; maxPET, maximum potential evapotranspiration [mm]; Bio1, annual mean temperature [°C]; Bio5, maximum temperature of warmest month [°C]; Bio6, minimum temperature of coldest month [°C]; Bio14, precipitation of driest month [mm]; Significance codes: ***0 < p ≤ 0.001; **0.001< p ≤ 0.01; *0.01 < p ≤ 0.05; (.) 0.05 < p ≤ 0.1.

Correlation table Model 1.

|  | relElev | Bio1 | Bio5 | Bio6 | maxPET | relElev2 | Bio1² | Bio5² | Bio6² | maxPET² |
| --- | --- | --- | --- | --- | --- | --- | --- | --- | --- | --- |
| relElev | 1 |  |  |  |  |  |  |  |  |  |
| Bio1 | -0.71 | 1 |  |  |  |  |  |  |  |  |
| Bio5 | -0.79 | 0.84 | 1 |  |  |  |  |  |  |  |
| Bio6 | -0.52 | 0.9 | 0.53 | 1 |  |  |  |  |  |  |
| maxPET | -0.37 | 0.23 | 0.43 | 0.01 | 1 |  |  |  |  |  |
| relElev² | 0.09 | -0.11 | -0.12 | -0.09 | -0.24 | 1 |  |  |  |  |
| Bio1² | 0.11 | -0.32 | -0.05 | -0.41 | -0.06 | 0.42 | 1 |  |  |  |
| Bio5² | 0.05 | 0.01 | 0.05 | -0.09 | 0.01 | 0.59 | 0.5 | 1 |  |  |
| Bio6² | 0.12 | -0.44 | -0.07 | -0.61 | -0.01 | 0.17 | 0.86 | 0.15 | 1 |  |
| maxPET² | -0.15 | 0.13 | 0.11 | 0.1 | -0.36 | 0.24 | -0.13 | 0.24 | -0.2 | 1 |

Correlation table Model 2 and Model 3.

|  | relElev | Bio1 | Bio5 | Bio6 | maxPET | Bio14 | relElev² | Bio1² | Bio5² | Bio6² | maxPET² | Bio14² |
| --- | --- | --- | --- | --- | --- | --- | --- | --- | --- | --- | --- | --- |
| relElev | 1 |  |  |  |  |  |  |  |  |  |  |  |
| Bio1 | -0.74 | 1 |  |  |  |  |  |  |  |  |  |  |
| Bio5 | -0.77 | 0.86 | 1 |  |  |  |  |  |  |  |  |  |
| Bio6 | -0.64 | 0.96 | 0.69 | 1 |  |  |  |  |  |  |  |  |
| maxPET | -0.35 | 0.22 | 0.32 | 0.13 | 1 |  |  |  |  |  |  |  |
| Bio14 | -0.05 | 0.17 | 0.1 | 0.16 | -0.23 | 1 |  |  |  |  |  |  |
| relElev² | 0.14 | -0.08 | -0.12 | -0.06 | -0.32 | 0.01 | 1 |  |  |  |  |  |
| Bio1² | 0.1 | -0.37 | -0.04 | -0.51 | -0.06 | 0.01 | 0.41 | 1 |  |  |  |  |
| Bio5² | 0.11 | 0 | -0.06 | 0.01 | -0.19 | 0.22 | 0.59 | 0.52 | 1 |  |  |  |
| Bio6² | 0.12 | -0.49 | -0.08 | -0.66 | 0 | -0.1 | 0.2 | 0.92 | 0.18 | 1 |  |  |
| maxPET² | -0.01 | -0.08 | -0.18 | 0.02 | -0.63 | -0.17 | 0.24 | -0.16 | 0 | -0.18 | 1 |  |
| Bio14² | -0.17 | 0.15 | 0.15 | 0.1 | 0.11 | 0.79 | -0.12 | -0.07 | -0.01 | -0.08 | -0.19 | 1 |

Best models (Model 1).

| Intercept | P | maxPET | P | maxPET² | P | relElev | P | relElev² | P | Bio1 | P | Bio1² | P | Bio5 | P | Bio5² | P | Bio6 | P | Bio6² | P | AICc | delta | $\text{R}_{\text{p}}^{\text{2}}$ |
| --- | --- | --- | --- | --- | --- | --- | --- | --- | --- | --- | --- | --- | --- | --- | --- | --- | --- | --- | --- | --- | --- | --- | --- | --- |
| 1.292 | *** | -1.297e-02 | *** | -3.622e-04 | *** |  |  | -7.440 | *** |  |  |  |  | 4.494e-02 | ** | -3.997e-03 | * | -3.220e-02 | *** | -1.486e-03 | ** | -240.7 | 0 | 0.41 |
| 1.255 | *** | -1.328e-02 | *** | -3.774e-04 | *** |  |  | -7.791 | *** |  |  | -3.995e-03 | *** | 3.826e-02 | ** |  |  | -2.480e-02 | *** |  |  | -240.0 | 0.71 | 0.41 |
| 1.240 | *** | -1.263e-02 | *** | -3.603e-04 | *** |  |  | -7.270 | *** |  |  | -3.301e-03 | ** | 3.629e-02 | ** | -2.326e-03 |  | -2.343e-02 | ** |  |  | -239.7 | 1.08 | 0.41 |

Best models (Model 2).

| Intercept | P | maxPET | P | maxPET² | P | relElev | P | relElev² | P | Bio1 | Bio1² | P | Bio5 | Bio5² | P | Bio6 | P | Bio6² | P | Bio14 | P | Bio14² | AICc | delta | $\text{R}_{\text{p}}^{\text{2}}$ |
| --- | --- | --- | --- | --- | --- | --- | --- | --- | --- | --- | --- | --- | --- | --- | --- | --- | --- | --- | --- | --- | --- | --- | --- | --- | --- |
| 0.9926 | *** |  |  | -9.25e-05 | * | 1.482 | *** | -8.197 | *** |  | 2.87e-00 | . |  | -7.80e-03 | ** | 2.95e-02 | * |  |  | 6.645e-03 | *** |  | -215.7 | 0 | 0.48 |
| 1.124 | *** | -4.75e-03 | . | -2.03e-04 | ** | 0.6527 | ** | -7.911 | *** |  |  |  |  | -5.35e-03 | ** |  | * |  |  | 5.958e-03 | *** |  | -215.6 | 0.13 | 0.48 |
| 1.122 | *** | -4.47e-03 | . | -2.00e-04 | ** | 0.906 | ** | -7.863 | *** |  |  |  |  | -5.54e-03 | ** | 1.15e-02 |  |  |  | 5.857e-03 | *** |  | -215.4 | 0.38 | 0.48 |
| 1.031 | *** |  |  | -1.18e-04 | ** | 1.14 | *** | -7.675 | *** |  |  |  |  | -5.43e-03 | ** | 1.25e-02 |  |  |  | 6.581e-03 | *** |  | -214.9 | 0.88 | 0.48 |
| 0.9902 | *** |  |  | -9.58e-05 | * | 1.445 | *** | -8.021 | *** |  |  |  |  | -6.06e-03 | ** | 2.90e-02 | * | 9.17e-04 |  | 6.656e-03 | *** |  | -214.8 | 0.89 | 0.48 |
| 1.027 | *** |  |  | -1.16e-04 | ** | 0.8794 | *** | -7.706 | *** |  |  |  |  | -5.24e-03 | ** |  | ** |  |  | 6.749e-03 | *** |  | -214.7 | 0.99 | 0.47 |
| 1.065 | *** | -3.10e-03 |  | -1.55e-04 | * | 1.134 | ** | -8.186 | *** |  | 2.16e-03 |  |  | -7.30e-03 | ** | 2.46e-02 | . |  |  | 6.128e-03 | *** |  | -214.7 | 1.07 | 0.48 |
| 1.074 | *** | -3.40e-03 |  | -1.65e-04 | * | 1.159 | ** | -8.034 | *** |  |  |  |  | -5.94e-03 | ** | 2.24e-02 |  | 5.94e-04 |  | 6.080e-03 | *** |  | -214.0 | 1.78 | 0.48 |
| 1.114 | *** | -5.09e-03 | . | -2.17e-04 | ** | 0.6479 | ** | -7.819 | *** |  |  |  |  | -5.26e-03 |  |  |  | -2.57e-04 |  | 5.824e-03 | *** |  | -213.9 | 1.81 | 0.48 |
| 0.9156 | *** |  |  |  | ** | 1.607 | *** | -8.899 | *** |  | 3.97e-03 | * |  | -8.19e-03 | *** | 3.52e-02 | ** |  |  | 6.840e-03 | *** |  | -213.9 | 1.88 | 0.48 |

Best models (Model 3).

| Intercept | P | maxPET | P | maxPET² | P | relElev | P | relElev² | P | Bio1 | P | Bio1² | P | Bio5 | P | Bio5² | P | Bio6 | P | Bio6² | P | AICc | delta | $\text{R}_{\text{p}}^{\text{2}}$ |
| --- | --- | --- | --- | --- | --- | --- | --- | --- | --- | --- | --- | --- | --- | --- | --- | --- | --- | --- | --- | --- | --- | --- | --- | --- |
| 1.417 | *** | -1.613e-02 | *** | -4.611e-04 | *** |  |  | -8.643 | *** |  |  |  |  | 5.198e-02 | * |  |  | -4.123e-02 | * | -2.342e-03 | ** | -177.4 | 0 | 0.39 |
| 1.353 | *** | -1.517e-02 | *** | -4.474e-04 | *** |  |  | -9.361 | *** |  |  |  |  |  |  |  |  |  |  | -8.647e-04 | * | -176.5 | 0.96 | 0.38 |
| 1.359 | *** | -1.534e-02 | *** | -4.547e-04 | *** |  |  | -8.826 | *** |  |  | -2.347e-03 | * |  |  |  |  |  |  |  |  | -176.4 | 1.00 | 0.38 |
| 1.38 | *** | -1.570e-02 | *** | -4.654e-04 | *** |  |  | -8.451 | *** |  |  |  |  |  |  | -2.747e-03 |  |  |  | -8.483e-04 | * | -176.2 | 1.25 | 0.38 |
| 1.4265 | *** | -1.636e-02 | *** | -4.704e-04 | *** |  |  | -8.172 | *** |  |  |  |  | 4.725e-02 | * | -1.651e-03 |  | -3.715e-02 | * | -2.182e-03 | ** | -175.9 | 1.51 | 0.39 |
